# Geothermal food dehydrator system, operation and sensory analysis, and dehydrated pineapple quality

**DOI:** 10.1002/fsn3.3249

**Published:** 2023-02-08

**Authors:** Eduardo Pérez‐González, Patricia Severiano‐Pérez, Héctor M. Aviña‐Jiménez, Olga Del C. Velázquez‐Madrazo

**Affiliations:** ^1^ Instituto de Ingeniería Universidad Nacional Autónoma de México Mexico City Mexico; ^2^ Laboratorios de Evaluación Sensorial y Microbiología, Departamento de Alimentos y Biotecnología, Facultad de Química Universidad Nacional Autónoma de México Mexico City Mexico

**Keywords:** food dehydration, geothermal energy, microbiological analysis, physicochemical characteristics, sensory profile

## Abstract

Food dehydration is a preservation technique that guarantees its supply. Food like vegetables and fruits are traditionally dehydrated with natural gas or solar energy, however, this work demonstrates the feasibility of doing it with energy from a geothermal power plant in Nayarit, Mexico. Different species of pineapple (Miel, Cayenne, and Esmeralda) were dehydrated at different temperatures from 64 to 80°C and the safety of the product was subsequently verified, for these aerobic mesophiles (<230 ufc/g), total coliforms (<0.3 s.m.), molds and yeasts (<120 v.e.), and salmonella spp (Absent in 25 g), and results were obtained within the proposed specifications, which were generated taking as reference the national and international guidance standards. A sensory evaluation, a modified Flash Profile (mFP), was carried out with a group of judges trained in descriptive methodology, since a better consensus of responses was obtained, thus demonstrating the usability of mFP for food dehydration. The studies of pineapple allowed the evaluation of production with the DGA 200 technology, and the microbiological standards, as well as sensory and physicochemical parameters, were considering just to verify that product is suitable for human consumption. The technology is a system that takes advantage of the heat of the earth, with which it is possible to work 7 days a week or the entire pineapple season. Physicochemical changes caused by its dehydration with respect to the content of vitamin C, carbohydrates, and dietary fiber in the three species of dehydrated pineapple were measured. In the fresh samples, an average concentration of vitamin C 9 mg/100 g, carbohydrates 11.6 g sugar/100 g, and dietary fiber 0.96% were measured. The dehydrated samples presented an average value of vitamin C of 95 mg/100 g, carbohydrates 72.6 g sugar/100 g, and dietary fiber 8.6%, these results were similar to Mühlbauer and Müller, 2020, *Drying atlas, drying kinetics and quality of agricultural products*, Elsevier.

## Graphical abstract

Food dehydration is a preservation technique that guarantees its supply. Different species of pineapple (Miel, Cayenne, and Esmeralda) were dehydrated at different temperatures from 64 to 80°C. Microbiological analysis, physicochemical characteristics, and sensory profile.

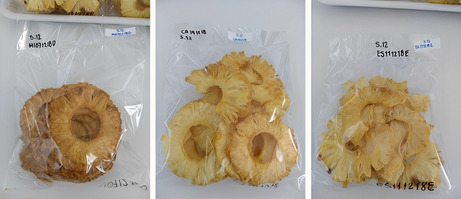

## 1 INTRODUCTION

Geothermal food dehydration is a process that allows food preservation without greenhouse gas emissions. Different prototypes have been developed since the last century, but very few have been scaled up to industrial production, which has been associated with the lack of spatial homogeneity of hot air, and to solve this, dehydrators have been designed with numerical simulation in Computational Fluid Dynamics, (Daza‐Gómez et al., 2022) and finally microbiologically and sensorially validate this product (Takougnadi et al., 2020). This work focuses on the evaluation of the product obtained with this dehydrator, reserving its numerical validation a follow‐up paper.

Geothermal energy is a renewable energy with a load factor, average in Mexico, >62% (Gutiérrez‐Negrin et al., 2020). It is classified by the amount of temperature or enthalpy: high, medium, or low (Iglesias et al., 2015). High enthalpy is used for the generation of electrical energy, while medium and low enthalpy are used for industrial, agricultural, and service applications grouped under the generic name of Direct Uses; the latter being developed with medium and low enthalpy geothermal energy.

To preserve food for longer, among the various methods for microbial control, the most widely used is heat. High temperatures denature the enzymes of microorganisms, resulting in a three‐dimensional structural change in said proteins and causing their inactivation.

Therefore, food dehydration must be carried out at temperatures higher than 45°C, thus guaranteeing its safety. The advantage of the DGA 200 compared with solar dehydrators (which also use renewable energy) is only the temperature inside the oven can be kept constant 24 h a day, 7 days a week, a feature that also offers advantages in the quality of the pineapple (López‐Cerino et al., 2018).

In Mexico, there is no regulation that establishes microbiological quality criteria for dehydrated fruit, so the microbiological guidance specifications used in Mexico for onion (NMX‐F‐233) and dehydrated garlic (NMX‐F‐250‐S‐1980) were used, as well as international standards (Ministerio de Salud, 2003; Ministerio de Saúde Brasil, 1978; Ministerio de la Protección Social de Colombia, 2011; Gilbert et al., 2000) and technical guides for dehydrated fruits and vegetables, from which, and based on previous studies carried out by this working group (the results are not shown), the following safety criteria is proposed for dehydrated pineapple (see Table S1).

Pineapple (*Ananas comosus* L.) is a very aromatic fruit with an oval to cylindrical shape, its size and weight can vary from 1.5 to 2.8 kg; their skin is characterized by being rough and thick. It is a multiple fruit (sorosis) consisting of a crown and a fleshy axis or heart, from which between 100 and 200 individual flowers start that merge with each other during the development of the fruit and that constitute the eyes of green tones and yellows on the shell. When the fruit is ripe, its pulp has an intense yellow color, flavors that go from sweet to acid and with soft to intense odor, very peculiar to this fruit.

Another of its main characteristics is the content of different volatile compounds in small amounts and complex mixtures, dietary fiber, minerals, and vitamins that offer a series of health benefits that include: reducing the risk of diabetes, colon cancer, cerebra‐vascular diseases, regulating emotional stability and strengthening the bone growth, antimicrobial activity, healing bowel movement and gastrointestinal function, anti‐inflammatory, antioxidant activity, monitoring nervous system function and digestion improvement, and cardioprotective agent (Hossain, 2016; Maimunah et al., 2020).

Sensory evaluation is defined as the scientific discipline used to measure, analyze, and interpret human reactions resulting from a stimulus caused by the consumption of a certain product. These studies are divided into two: analytical tests and affective or hedonic test (Severiano‐Pérez, 2019). Most of disciplines that support sensory studies are mainly statistics and psychology.

These types of studies are very important in food industry, since they allow solving problems related to the quality of food, involving odor, flavors, colors, and textures through the senses; therefore, it is necessary to have a group of judges, called a sensory panel, with training so that the results are more reliable, precise, exact, and representative (Baldán et al., 2021; Väkeväinen et al., 2020).

There are several studies that use the color and texture parameters to determine physical changes in dehydrated fresh pineapple samples, since they are parameters that allow these aspects to be correlated with the organoleptic properties of the fruit and thus establish the quality of the product (Chikpah et al., 2022).

For instrumental analysis of color in food, the CIE system is suitable (Carmona‐López, 2016). The color indicators L*, a*, b*, are used to describe the changes in coloration (Carmona‐López, 2016; Chikpah et al., 2022; Horuz & Maskan, 2015; Serdar & Ayse‐Vildan, 2016). The CIELab conditions are given under a scale in a three‐dimensional space where L* is luminosity (L* = 0 black yields and L* = 100 white yields), a* goes from red to green (a*, negative values indicate green while positive values indicate red), b* is the gradient of blue (b*, negative values indicate blue and positive values indicate yellow) (Carmona‐López, 2016). Studies that have been carried out on this subject are related to the changes in color in foods subjected to various dehydration processes (osmotic, high‐pressure processing, hot air, and microwaves) (Horuz & Maskan, 2015; Marconi et al., 2017; Snehasis et al., 2015), kinetics of pineapple puree color degradation after heat treatment (Chikpah et al., 2022), as well as in shelf‐life studies (Calligaris et al., 2022).

Texture is a multidimensional characteristic in dehydrated fruits, and its main object of study is the rehydration capacity, studies that have generally been done in onion, hawthorn, and pineapple (Roman et al., 2020; Serdar & Ayse‐Vildan, 2016; Simao et al., 2021). Hardness or firmness determination is important to know about maturity degree when fresh or the hardness they acquire after a process such as dehydration (Montero‐Calderón, 2010), which is measured with a small probe, with which a force is applied. Which is measured in the texturometer and which allows graphing the relationship of said force and the penetration distance or time, counting from when the probe touches the surface of the object and until it detaches from it (Carmona‐López, 2016). In this way, the production capacity and quality of the dehydrated pineapple with the use of the DGA 200 technology were evaluated. The methodology used for its dehydration is presented in Table S10, and the logic diagram of the research is in Figure S10.

## 2 MATERIALS AND METHODS

### 2.1 Samples

Pineapples, which were dehydrated in the DGA 200, are originally from the state of Nayarit, where the Cayenne, Miel, and Esmeralda species can be found. Its classification according to maturity degree was made based on the document PC‐029‐2005 “Specifications for the Use of the Official Brand México Calidad Suprema en Piña”, which establishes the guidelines of the official brand, Calidad Suprema, which is owned by the Government of Mexico, and is granted to the Secretariat of Agriculture, Livestock, Rural Development, Fisheries and Food (SAGARPA, 2005; see Figure S7 and Table S9).

Pineapples were purchased at the end of November 2018 and were selected according to specifications in *NMX‐FF‐028* standard (Gobierno de México, 2008). Maturity level which they were purchased was No. 2 (see Table S9), thus giving the opportunity to mature them and homologate their level for processing in the dehydrator. It is worth mentioning that manufacture of pineapple dehydration *NOM‐251‐SSA1‐2009* was followed (Gobierno de México, 2009).

This fruit was chosen for its high content of sugar, vitamin C (ascorbic acid or AA) and an amount of moisture, fruit characteristics that demand a large amount of energy for dehydration (Iglesias & Chirife, 1982) but that make it a delicate food due to its easy caramelization of sugar and loss of AA.

### 2.2 Pineapple dehydration

In Table S10, steps that make up the process of preparing dehydrated pineapple are detailed, from its selection to its packaging. Once the production batches of the dehydrated pineapple were obtained, their microbiological, physicochemical, and sensory quality were evaluated.

### 2.3 Microbiological analysis

For preparation and dilution samples, Mexican Standard NOM‐110‐SSA1‐1994 was followed. For the determination of aerobic mesophylls, Official Mexican Standard NOM‐092‐SSA1‐1994 was used; total and fecal coliforms were determined following Official Mexican Standard 112‐SSA1‐1994; molds and yeasts according to NOM‐111‐SSA1‐1994. On the other hand, *Salmonella* spp was determined and by means of rapid Compac Dry® tests, it should be noted that it is an alternative method that has international validation by AOAC, AFNOR, or ISO, as established by NOM‐210‐SSA1‐2014. Products and services. Microbiological Test Methods. Determination of indicator microorganisms. Determination of pathogenic microorganisms, in Section 3.15.

### 2.4 Physicochemical analysis

As a dehydration control, the two physicochemical characteristics that were measured were water activity (*a*_w_), which was determined with a MS21000 Handheld water activity meter, and Total Soluble Solids (°Brix), which were determined with a refractometer according to Official Mexican Standard NMX‐F‐112‐2010.

In order to know the characteristics of the three varieties of pineapple, samples of pineapple Miel, Esmeralda and Cayenne, two of them; It is worth mentioning that all the samples from Cayenne 1 to 5 belong to a general batch, but they were dehydrated in different days and under different conditions, presenting similar characteristics, however, Cayenne 2 was selected because it dehydrated at an intermediate temperature to that of the rest and for its sensory similarity with the Esmeralda pineapple; while Miel pineapple was selected as a case of interest for being a totally different sample from the others, as well as for its high dehydration temperature.

To take the main characteristics of dehydrated pineapple as a reference, the AA content was determined through the AOAC 1990 697.21 method, crude protein, using the AOAC 2015 2001.11 method, carbohydrates, using the DNS reducing sugars method and total sugar phenol sulfuric method, acidity, AOAC method 2015 940.15 and dietary fiber, AOAC method 985.54.

### 2.5 Sensory evaluation, modified flash profile

The modified flash profile (mFP) is carried out with a group of judges trained in descriptive methodology, since a better response consensus is obtained, by virtue of the fact that it allows generating a joint list of attributes for dehydrated pineapple (Väkeväinen, y otros, 2020). An evaluation panel was made up of 13 panelists (21–47 years old students, 8 women and 5 men, students of the Faculty of Chemistry, UNAM), who already had 1 year of experience as judges trained in conventional descriptive analysis to evaluate dehydrated foods, such as tomato, guava, mango, and papaya. The evaluations were carried out in the Sensory Evaluation Laboratory of the annex of the 4D Laboratory, building A, in the Faculty of Chemistry of the UNAM, currently complies with the ISO, 2007 standard.

In the first session, judges were presented with four samples of dehydrated pineapple (Esmeralda, Miel, and two samples of Cayenne), and the judges were asked to generate the sensory attributes of appearance, smell, taste, texture, and aftertaste for each one of the samples. In the second session, a consensus list of attributes was prepared (eliminating synonyms, ambiguous, or affective terminology) and the scales on which each attribute would be evaluated were defined. And finally, the seven batches of dehydrated pineapple object of this study were evaluated. For the evaluation of the samples, a dehydrated pineapple slice was placed; all samples were identified with three‐digit codes and evaluated at room temperature. The judges were also provided with a glass of water and some whole grain cookies to rinse the palate between each sample (Figure S8). Sensory assessment sessions and questionnaires were designed using FIZZ software (Biosystems, version 2.51c, Acquisition and Judge modules). The scale to measure the intensity of the attributes was a 9‐point scale, where 1 represents the minimum intensity of the stimulus and 9 the maximum.

### 2.6 Instrumental analysis: Color and texture

For the color analysis, the color parameters were measured at room temperature, using the CIE, L*, a*, b* system, with a Minolta CM‐3600d spectrophotometer. The dehydrated pineapples were wrapped in kleen pack paper, and the equipment was fitted with a small viewing area, illuminant D65 and six replicates were evaluated per sample.

Excluded Specular Component was selected because it allows correlating the measurement made by the human eye and the instrument, therefore, when evaluating the color, it considers the brightness and texture (appearance) of the sample, which allows greater discrimination by the team. The detector that acts as an observer is in all analyses at 10° (Escamilla‐Morón, 2006).

In texture analysis, the puncture analysis was used, which is responsible for measuring the force required to introduce a probe into a food and measure its hardness or firmness (Bourne, 2002; Sahin & Gülüm, 2009). A 2 mm diameter conical probe (P/2 N) was used, with the TA.XT2i equipment, Texture Analyzer Stable Micro Systems, Texture Expert EXCED® software at a force of 0.7 N and a speed of 2 mm/s, at room temperature, six repetitions per sample were evaluated.

### 2.7 Statistical analysis

For statistical analysis of data, XLSTAT Microsoft Excel® software (XLSTAT version 2020.2.2, Addinsoft) was used.

Data from *a*_w_ and °Brix were analyzed with one‐way ANOVA (Analysis of Variance) at *α* = 0.05 and minimum significant difference that allowed determining if there was a statistically significant difference between the samples.

mFP statistical analysis data were performed with the generalized procrustes analysis (GPA) multivariate method. The GPA is considered an exploratory analysis of multivariate data and provides a graphical interpretation of the distances between samples, which is called sensory attribute space. This two‐dimensional analysis uses translation, rotation, and scaling to obtain the mean position of the products (Terhaag & Benassi, 2010).

To evaluate the consensus of the panel and visualize the relative sensory positioning of attributes and products, principal component analysis (PCA) is used (Väkeväinen et al., 2020).

To simultaneously process the results of the different analyses (texture, sensory color), the multiple factor analysis was carried out, which allows the simultaneous analysis of various tables of variables, and obtain results, especially graphs, that allow studying the relationship between observations, variables, and tables. Within a table, the variables must be of the same type (quantitative or qualitative), but the tables can be of different types (XLSTAT, 2021) to simultaneously evaluate several sensory attributes of appearance and texture with the data obtained from the instrumental evaluation of color and texture.

## 3 RESULT AND DISCUSSION

### 3.1 Pineapple dehydration

Pineapple (Figure S1) was dehydrated with geothermal energy from the separation platform PS‐02 of the Geothermal Power Plant Domo San Pedro. The DGA 200 represents an installed capacity of 0.53 MWt, uses 3676 GWh/year, equivalent to ceasing to emit 338.55 t CO_2_/GWh, or what is equal to 1.25 Mt CO_2_/year.

### 3.2 Microbiological analysis

The first analysis carried out on the batches was the microbiological one (see Table S3). It can be observed that the values for aerobic mesophylls, total coliforms, molds, and yeasts and *Salmonella* spp are within the proposed specifications, dehydration conditions used in the seven batches allowed obtaining samples with good microbiological quality.

Aerobic mesophiles are between half and one logarithm below the limit, which speaks of good raw material quality and good hygiene and sanitation practices. It is worth mentioning that in previous works (Carmona‐López, 2016) a standardized procedure was designed for conditioning and sanitizing pineapple before subjecting it to drying; Mesophilic results indicate that this procedure works well. Regarding coliforms, they are not detected in any of the samples, even with a method with high sensitivity, such as the most probable number, this result is especially important because coliforms are indicators of contamination due to unsanitary practices, as well as the quality in the efficiency of the processes of elimination of microorganisms. Facts that are not detected in the analysis is an indicator of good manufacturing practices and an efficient process for reducing microbial load. Molds and yeasts are indicators of hygienic practices and environmental contamination. Results are one log below the limit in five of the seven batches, the other two batches are less than half the limit. It is the indicator with which it is necessary to pay more attention, but it can be affirmed that they are under control and that the product has good microbiological quality. *Salmonella* spp was absent in all samples, as required by food standards.

Microbiological growth from the point of view of the *a*_w_ content, did not present any problem, since for the seven samples results below 0.6 were obtained (see Table S4), which is considered by the Secretary of Health of Mexico, the United States FDA and the European Economic Community as a critical point to determine whether a food is stable or not. Another important aspect to mention is the process temperatures, which as can be seen, are higher than the microbiological growth limit mentioned in Section 1.2.

### 3.3 Physicochemical analysis

In Table S4, the *a*_w_ and °Brix results of the 7 batches of dehydrated pineapple are shown, in which the samples with the lowest *a*_w_ were Cayenne 4, 5 and Miel, which were the samples that dehydrated the highest temperature (70.0, 80.0, and 78.4°C, respectively; Figure S9). Differences between each one of the samples have to do with the dehydration temperature and the variety of the pineapple, since it is reported that the cayenne pineapple has 85.61% (Sirijariyawat & Charoenrein, 2012) of humidity while the Miel has 85%. Another parameter evaluated was the °Brix, observing that the samples with the lowest °Brix was Cayenne 5, and the one with the highest °Brix was Cayenne 3.

As can be seen, the greater the relationship between the °Brix content and the amount of water available in the feed (*a*_w_), they coincide with that reported by López‐Cerino et al. (2018), which says that the greater the amount of dissolved sugars, the greater the thermal energy necessary for their dehydration, so if the process conditions were similar (temperature and time) for the seven samples (Table S2), it was to be expected that those samples with high °Brix index had higher *a*_w_.

Table S5 presents the physicochemical results of the three selected samples, of which a shelf‐life study was also carried out, but the results are reserved to be presented in another publication.

Pineapple as an important source of bioactive compounds that include antioxidants such as AA, flavonoids, phenolic compounds and pectins, characterize it as an important food for nutrition (Ghasemi et al., 2009; Kumar et al., 2021; Montero‐Calderón, 2010); and of these bioactive compounds, the one of greatest interest is AA as it is one of the volatile compounds that is degraded by various variables such as pH, the presence of enzymes, oxygen, metallic catalyzers, light, and temperature (Santos & Silvia, 2010), the latter being a relevant aspect for hot air dehydration processes.

From evaluated samples, Miel presented a high content of AA despite having been dehydrated at a higher temperature, compared with the Esmeralda and Cayenne varieties, which presented a content lower than 55 mg/100 g, and it was to be expected because the time of exposure to heat was 15 h, while for Esmeralda and Cayenne 2 the exposure to high temperature was 20 and 18 h, respectively; The quantity of pineapple that was dehydrated was also greater, 20 and 36 kg of pulp, respectively. Similar results are reported for pineapple dehydrated at 60°C, slices 10 mm thick, and dehydration for 13 h, with a final AA content of 101 mg/100 g (Mühlbauer & Müller, 2020); and correlating this phenomenon in tomato with slices of 11 mm at a temperature of 6°C and exposure time between 16 and 20 h, a loss of AA between 30% and 40% was recorded (Khazaei et al., 2008), the above also coincides with what was reported by 40 studies of various dehydrated foods, where 43% lost between 21.8% and 53% of AA under similar dehydration conditions (60°C) (Červenka et al., 2018). It should be mentioned that although pineapple is not within the aforementioned studies, it is characterized as an important source of bioactive compounds that include antioxidants such as ascorbic acid, flavonoids, phenolic compounds, and pectins that are important for nutrition (Ghasemi et al., 2009; Kumar et al., 2021; Montero‐Calderón, 2010). Unfortunately, it was not possible to measure flavonoids and phenolic compounds. Some authors show that the processing of fruits and vegetables decreases the content of their bioactive compounds and their antioxidant potential; For example, boiled and baked tomatoes have a relatively small effect on phenols, AA, lycopene, and antioxidant potential, while frying them significantly reduces total phenols, AA, and lycopene (Fabani et al., 2020; Gorinstein et al., 2010), which is why it is considered that bioactive compounds are present in pineapple, since dehydrated pineapple was not processed at high temperatures, such as frying process temperatures, so it is important to perform analyses and measure flavonoids and phenolic compounds in future work.

Three of the samples reported similar amounts of protein and coincides with that reported in dehydrated pineapple (2.5%) (Gómez‐Rangel, 2019; Mühlbauer & Müller, 2020). On the other hand, cayenne pineapple was the one that presented the highest dietary fiber. Regarding the amount of carbohydrates that the dehydrated pineapple samples present, they are within a range of 69–77 g per 100 g of sample, which coincide with those reported in dehydrated pineapple (commercial products) with values of 65.7 g/100 g (Mühlbauer & Müller, 2020).

### 3.4 Sensory analysis, modified FP

Attributes selected to evaluate the profile of dehydrated pineapple are shown in Table S6.

In Figure S3, GPA results for taste and odor of the 7 batches of dehydrated pineapple are shown, the PCA of the results explains 80.23% of the variability of the samples. The F1 component explains 67.82% of the variability of the data, while the F2 component explains 12.41% of the variability; positively correlated to factor 1 and factor 2 is fresh pineapple, which is associated with flavor: fermented, salty, fresh, pineapple, sweet, and astringent; smell: citric, fermented, sweet, fruity, fresh, and pineapple, and aftertaste: astringent and blanched. Cayenne samples 5, 4, and 3 were correlated with the intensity of flavor and odor, flavor: sour and caramelized and odor: roasted. On the other hand, the samples of Cayenne 2, 1, Miel, and Esmeralda were negatively related to factor 1 and positively to factor 2 and were characterized by their caramel smell and bitter aftertaste.

For the batches that presented caramelized odor and bitter aftertaste, it coincides with high dehydration temperatures (more than 70°C), and they were the group with the highest sugar content. This is due to the modifications in the odor and flavors associated with the caramelization reaction. In dehydrated foods, fresh, green, and sweet flavor notes are sought (Carmona‐López, 2016), which are normally lost in dehydration due to working temperatures, and due to the sensitivity of compounds such as carotenoids and fatty acids. Consequently, the characteristic color of pineapple turns to a coloration that oscillates between brown/toasted and this is also attributed to the production of HMF (Zanoni et al., 1999).

Regarding the sugar content in pineapple, it is this characteristic that attributes to the fruit the quality of requiring a large amount of activation energy to be able to dehydrate it and it is this characteristic that led to the selection of pineapple as a product in this study for the dehydration study. The results for appearance, odor, taste, and aftertaste are presented below, Figure S2.

When carrying out the profile with a group of trained judges, the consensus of the judges is expected to be good (Väkeväinen et al., 2020), and this was observed in the pineapple evaluation (Figure S3), coinciding with what was reported in other studies where mFP was used.

### 3.5 Multiple factor analysis, correlation between sensory characteristics, and instrumental color and texture

To know the correlation between the sensory attributes of appearance and texture, as well as the color and texture measured instrumentally, a multiple factor analysis (MFA) was carried out, Figures S4 and S5. Four groups of variables were considered, of which two of them correspond to the instrumental parameters (texture and color) and the others are related to the sensory variables of appearance and texture.

Between the two factors of the MFA, factor 1 and factor 2, they explain 60.55% and 17.25% of the total variety in the results. In general, fresh samples are different from dehydrated samples.

Positively correlated to both components, fresh pineapple was found, which was correlated with the sensory attributes of appearance: fibrous and yellow color, and texture: cohesiveness, fibrous, rough, astringency, juicy, hardness, and chewiness; and with hardness and fracture measured instrumentally. Juiciness was expected to be correlated with fresh pineapple since this fruit has an average of 85% humidity (Mühlbauer & Müller, 2020), and for this reason it is considered a juicy fruit. As mentioned in Section 1.2, it is a multiple fruit, since it is constituted by the fusion of 100–200 individual flowers, integrating a fleshy axis or heart and these characteristics could influence the perceived fracturing; on the other hand, its centre is characterized by being astringent (Montero‐Calderón, 2010) and is associated with what was already mentioned in Section 3.3.

Fresh pineapple was hard and this was perceived both sensorially and instrumentally, which agrees with the studies on fresh pineapple by Montero‐Calderón (2010), it is likely that this attribute has been correlated with the fresh pineapple because the fresh slice was of 5 mm and when dehydrated it shrank up to 2 mm thick, this could influence the perception of hardness due two factors; the first is related to its high percentage of shrinkage, since there are studies that show that at high temperatures shrinkage decreases and vice versa (Horuz & Maskan, 2015;Roman et al., 2020; Serdar & Ayse‐Vildan, 2016), while the second factor is the porosity in the food, which is also related with its shrinkage, the greater the formation of pores, the smaller its volume reduction or change in dimensions, due to the elimination of the water contained in the pineapple (Simao et al., 2021).

On the other hand, the dehydrated samples were negatively correlated to component 1, which indicates that their sensory profile is different from that of the fresh samples. Cayenne 5, 1, and Miel, but only Cayenne 1 and Miel were positively correlated to component 2 and to the sensory attributes of appearance: toasted and brown tips, texture: crunchy, billability, and rough, as well as to component a* of the CIE Lab scale Serdar and Ayse‐Vildan (2016) established that, if the dehydration time is long, the L* and b* values decrease, which is directly reflected in dark colourations due to the loss of luminosity and yellow color, by color brown as reported in this work.

Cayenne 5 and 1 were dehydrated at 80 and 71.4°C, respectively, which caused the samples to appear toasted and with brown tips, this last unwanted characteristic in the dehydrated product, so it can be concluded that the samples pineapple of these two varieties should be dehydrated at temperatures below 70°C (between 55 and 65°C) to avoid this defect. The loss of pigmentation is related to oxidative damage to the tissue, which is more easily manifested in foods with high sugar content, being the sample of Cayenne 1 that presented 13.6 °Brix, and Cayenne 5, 10.7 °Brix, which although it was one of the smallest values of the seven batches, it was the one that was subjected to the highest temperature of all. Horuz and Maskan (2015) demonstrated that prolonged exposure of food to high temperatures increases pigment oxidation and enzymatic and non‐enzymatic browning.

Crunchy texture is expected in a dehydrated fruit (Carmona‐López, 2016), as well as a medium fracturability (7.9 N) to prevent the samples from breaking during packaging and subsequent storage, in this study only the samples dehydrated at more than 75°C presented high fracturability (>9.5 N), which caused some samples to break, but those dehydrated below 75°C presented lower fracturability (6.7 N), which allowed to keep the slice intact during storage. The structural mechanics indicates that the great potential of efforts generated in the cellular structure of the food is what will determine the degree of shrinkage, and in turn, this will determine its rehydration capacity. The stress potential depends on two factors, the first is the high temperatures, while the second is the long dehydration times under which the food is subjected. It has been determined for different foods that porosities after a dehydration process at high temperatures and prolonged exposure times generate more pores in the structure, which is related to a high fracturability in dehydrated products (Roman et al., 2020; Serdar & Ayse‐Vildan, 2016). As mentioned before, shrinkage and porosity are closely related to another quality that is sought in dehydrated foods, which refers to rehydration. Rehydration is an important property which allows understand the quality of the dehydration process. Serdar & Ayse‐Vildan, 2016, determined that the rehydration capacity is affected if the speed and temperature of the air decreases, this is mainly due to changes in the structure; if the surface of the food lacks pores, it means that the structure collapsed and will be reflected in its shrinkage, therefore, the penetration of water into the tissue in a rehydration process will be affected by the few cavities and pores present in the tissue. Similar results are reported by Abbasi et al., 2011; Kumar et al., 2021 and Riveros‐Gomez et al., 2022, who report better rehydration of the samples dehydrated at a higher temperature (70°C) compared with those that were dehydrated at a lower temperature (60 and 50°C).

The Cayenne 2, 3, 4, and Esmeralda samples negatively correlated to component 1 and 2, presented an appearance: homogeneous and a texture: sensorially measured adhesiveness and correlation with the components b*, h*, and C*, L* of the CIE scale Lab. These samples presented a yellow hue with an average value of h* of 70.92, 83.81, and 48.11, respectively; although they presented an adhesive sensory texture, this was low and did not represent a problem for the sample, since having a higher *a*_w_ (>0.5) than the Cayenne 5 and Miel samples (of the order of 0.48), they are less fractured than the samples Cayenne 5, 1, and Miel, presenting a texture that maintains its shape when packaged. Its homogeneity is an aspect desired by consumers who consider that this attribute together with the color of the food makes it more palatable (Carmona‐López, 2016). It should be mentioned that the yellow coloration in the Cayenne 2, 3, 4, and Esmeralda samples indicates a low oxidation in the pigmentation, as already mentioned, and that it coincides with the acid astringent taste from ascorbic acid, which has been shown to as long as caramelization reactions do not prevail, this compound will remain (Chikpah et al., 2022; Horuz & Maskan, 2015; Serdar & Ayse‐Vildan, 2016).

On the other hand, the Cayenne 1, 5, and Miel samples were negatively correlated to component 1 and positively to component 2, presenting color characteristics: dark brown, toasted and brown tips, and a texture: crunchy, fractured, and rough the color of these samples It was dark brown and that explains its correlation with the a* component, which indicates that the samples were subjected to a higher temperature, Miel and Cayenne 5, or a higher sugar content, which caused greater caramelization, in this case the Cayenne 3 sample it was the one with the highest sugar content, 12.83 °Brix.

Previous studies with different dehydrated fruits such as guava, mango, papaya, and even tomato, it was concluded that the dehydrated products with geothermal energy, sensorially speaking, retain attributes of the fresh material, among the characteristic attributes in general for dehydrated samples are the sweet notes, adhesive, flexible texture, characteristic smell and flavor of each fruit, gloss, and acid flavor (Carmona‐López, 2016).

## 4 CONCLUSION

The DGA 200 dehydrator allows pineapple dehydration with a microbiological quality that broadly complies with parameters established in this work. Although there are microbiological references of onion and dehydrated garlic in Mexico, they are not compared with pineapple, in principle because they are different foods, but mainly because garlic and onion have quite active antimicrobials (Benkeblia, 2004), On the contrary, the high content of moisture, sugar, and fiber represent a highly nutritious medium that favors microbial growth in fresh pineapple (Hossain, 2016). For this reason, special attention was paid to the process temperatures and the final *a*_w_; The microbiological results obtained, always well below the limits for dehydrated garlic and onion (which have antimicrobial substances), demonstrate the proper functioning of the dehydrator from a microbiological point of view and the safety of its products.

When the samples maintain a yellow color, it is due to the absence of caramelization, which is associated with oxidation due to high temperatures (above 75°C) and high sugar content (between 12 and 13 °Brix). Therefore, these aspects must be considered in pineapple dehydration; for example, if samples have a high sugar content and are dehydrated at 70°C, there will be caramelization; on the other hand, for samples with low sugar content (10 °Brix) subjected to 80°C for a long time, they will present the same caramelization phenomenon.

Texture plays an important role from the point of view in the perception that the consumer has, that it is pleasant, but at the same time it defines the shelf life of the product, since if it is fractured when it is packaged, it takes away its presentation and taste. However, the presence of this attribute is an indicator of the high porosity that it has and consequently its easy rehydration capacity that it will maintain, so it is important to define the final objective of the dehydrated pineapple and with it the final quality that will be given.

In this sense, the main nutritional characteristics present in dried pineapple represent an opportunity to replicate and scale the technology in other communities that are agricultural areas and with high potential in the use of geothermal energy.

Finally, it is recommended to consult the supplementary material to see details of the process flow diagram (Figure S6), as well as the thermodynamic calculations (Table S7) and (Table S8).

## FUNDING INFORMATION

The authors received no financial support for the research, authorship, and/or publication of this article.

## CONFLICT OF INTEREST STATEMENT

The authors declare that they have no conflict of interest to report.

## Supporting information


Data S1

Figure S1

Figure S2

Figure S3

Figure S4

Figure S5

Figure S6

Figure S7

Figure S8

Figure S9

Figure S10

## Figures and Tables

**FIGURE A1:**
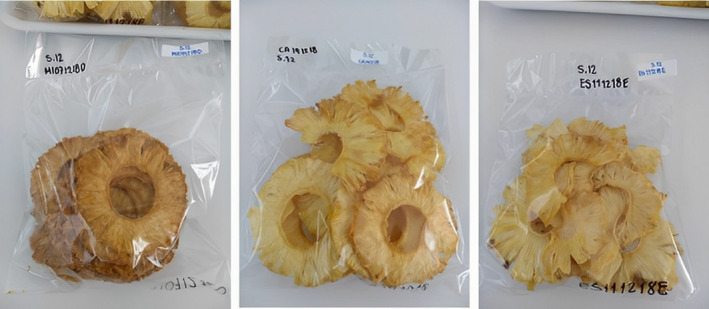
Dried pineapple, from left to right Miel, Cayenne 2, and Esmeralda.

**FIGURE A2:**
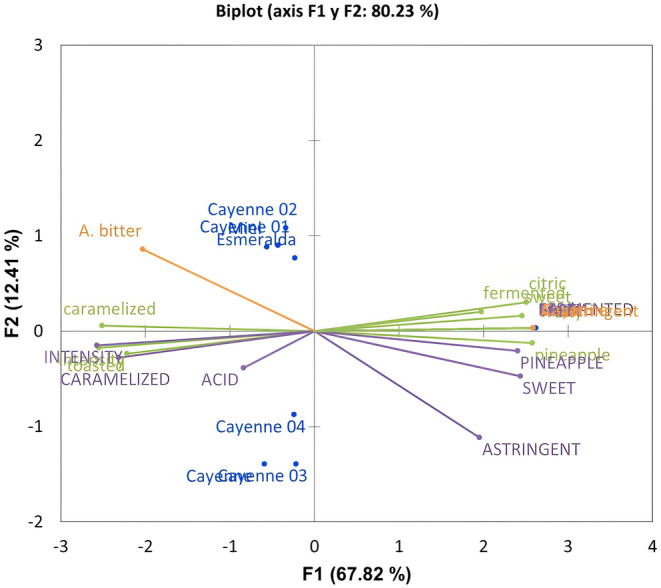
Principal component analysis from the odor and taste comparison for fresh and dehydrated pineapple.

**FIGURE A3:**
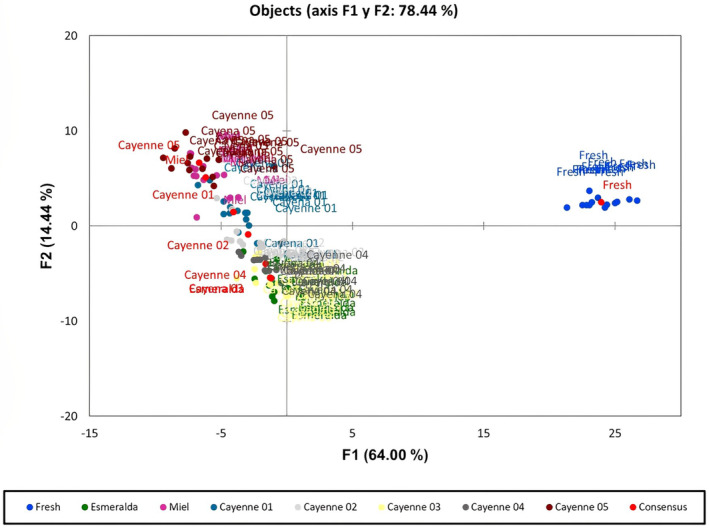
Consensus result of the seven pineapple samples.

**FIGURE A4:**
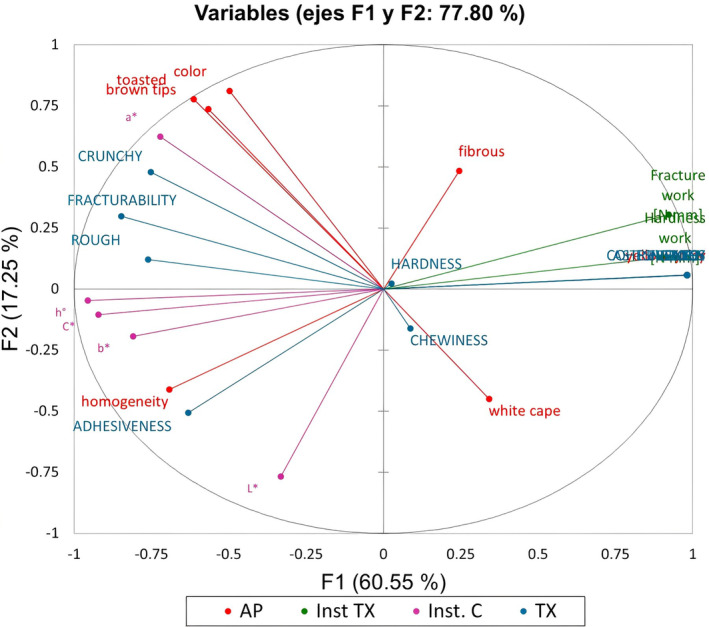
PCA mapping from Multiple Factor Analysis of the instrumental and sensory (appearance and texture) in dehydrated pineapple.

**FIGURE A5:**
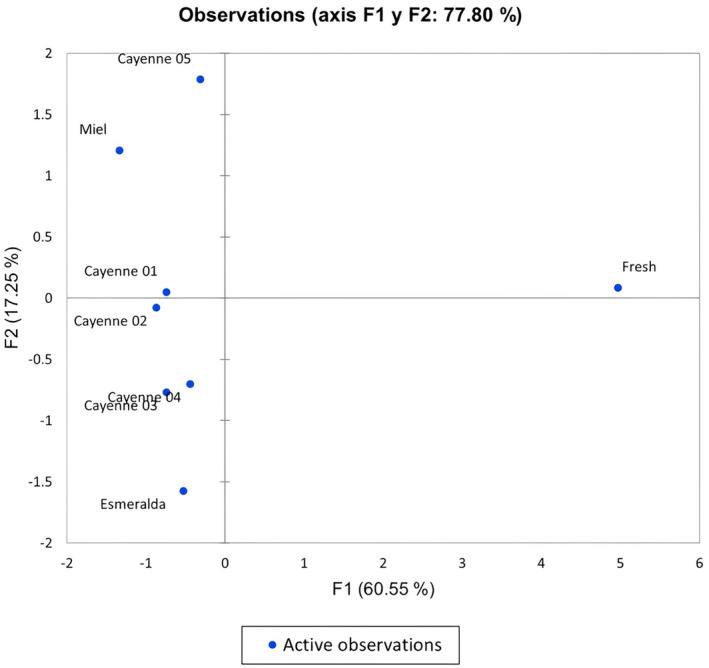
Dehydrated pineapple sensory space, obtained from Multiple Factor Analysis.

**FIGURE A6:**
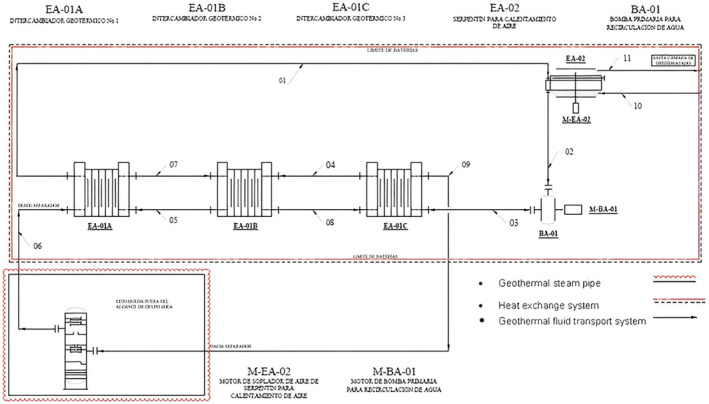
DGA 200 System Process Flow Diagram.

**FIGURE A7:**
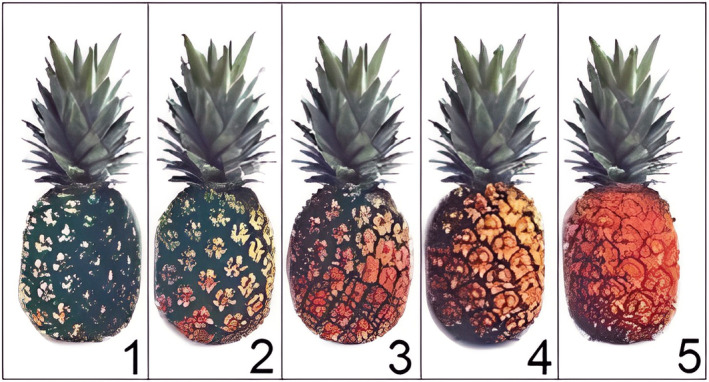
Table for the classification of pineapple according to external color (SAGARPA, 2005).

**FIGURE A8:**
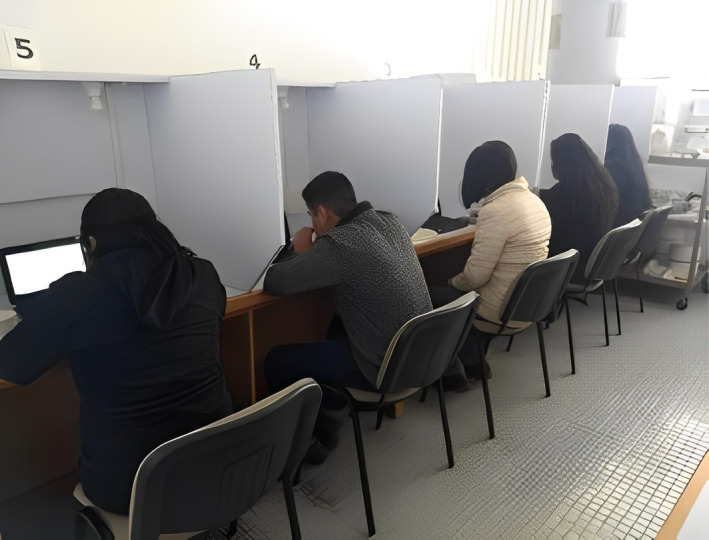
Sensory evaluation of dehydrated pineapple.

**FIGURE A9:**
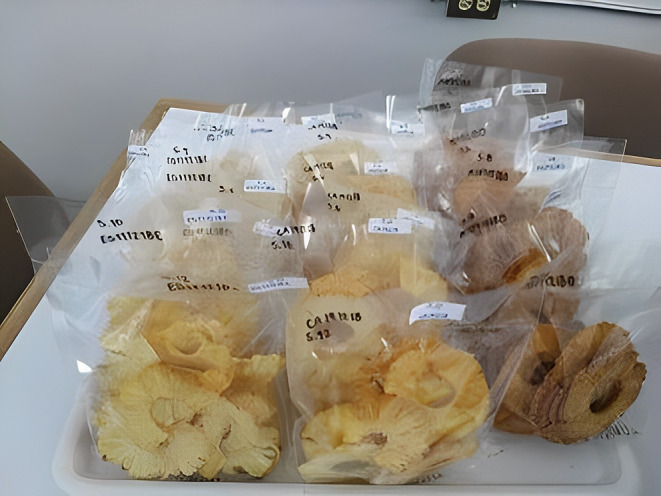
Different batches of dehydrated pineapple.

**FIGURE A10:**
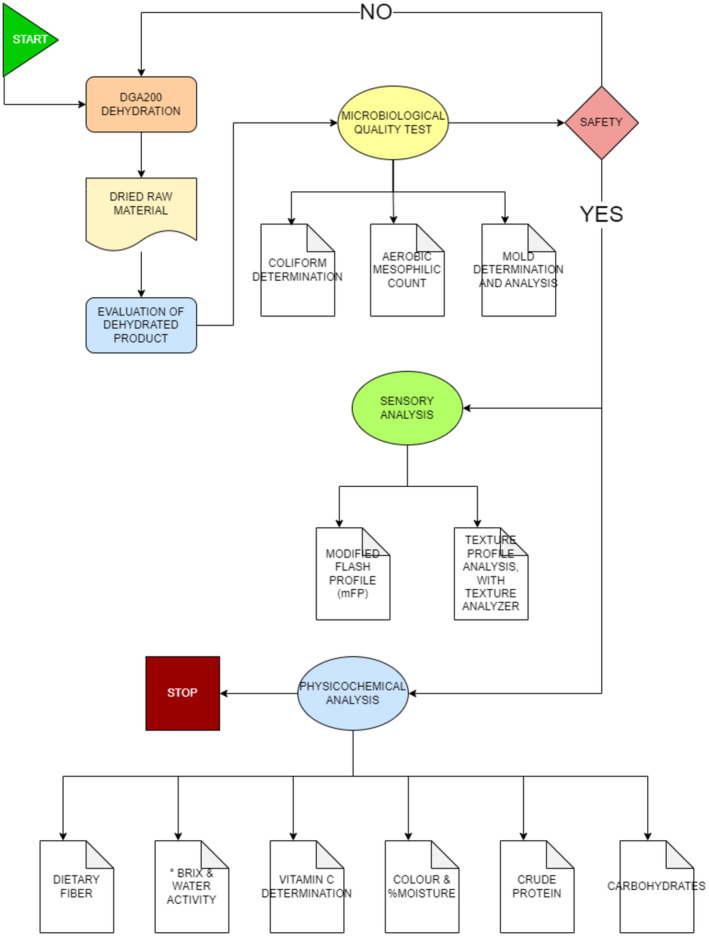
Logic diagram of the research.

## Data Availability

The data that support the findings of this study are available on request from the corresponding author.

## APPENDIX 1 DGA 200 SYSTEM

### 1.1

Moisture removal in food can be carried out by different methods; however, 85% of dehydrators in the industry are hot air and direct heating with temperature ranges between 50 and 400°C (of course this is general information, and the temperature does not go so high for food products; Mujumdar et al., 2010). The DGA 200 is a technological development conceived at the National Autonomous University of Mexico (UNAM), and it was installed and tested in the first private geothermal field in Mexico and its production capacity ranges from 150 to 200 dry kg/day, and for this reason, it is considered the first technological development of this nature; although there are already geothermal dehydrators in several countries that have this energy resource, most are developments at the prototype level and the quality of dehydrated foods has not been documented. Therefore, it was sought that the DGA 200 be at a level of commercial production under the quality standards demanded by the industry and at the same time meeting consumer expectations through a sensory analysis (Figures A1, A2, A3, A4, A5, A6, A7, A8, A9, A10; Tables A1, A2, A3, A4, A5, A6, A7, A8, A9, A10).

It could be thought that a food with a higher moisture content will represent a higher energy consumption for the process; however, there are studies that show that the content of dissolved solids influences the energy consumption required in the process, and pineapple is precisely one of the food that needs more energy to dehydrate it (Hossain et al., 2001; Simal et al., 2006); and for this reason, it was used as a reference feed in the validation of the DGA 200. Table A7 and Figure A6 show the thermodynamic states of the process and the process flow diagram, respectively, of the dehydrator system. Dehydration system is characterized by four subsystems (1) geothermal steam pipe, (2) heat exchange system, (3) geothermal fluid transport system, and (4) dehydration chamber, in Table A8 each one is defined.

**TABLE A1:** Guidance of bacterial pathogens (the Hazard) specifications for dehydrated pineapple.

Hazard	Maximum permissible
Aerobic colony counts or *aerobic mesophylls*	500 cfu/g
*Coliforms* organisms	25 cfu/g
Yeasts and moulds	300 cfu/g
*Staphylococcus aureus* (coagulase‐positive)	<100 cfu/g
*Salmonella* spp	Absent in 25 g
*Escherichia coli*	Negative

Abbreviation: cfu, colony forming unit.

**TABLE A2:** Information on the seven batches processed in the DGA 200.

Pineapple	Production, lot	kg, dry matter	Drying time, h	Temperature, °C
Miel	08122018	14.300	15	78.4
Esmeralda	11122018	20.078	20	67.1
Cayenne 1	15122018	31.528	18	71.4
Cayenne 2	19122018	36.078	18	72.0
Cayenne 3	09122018	10.482	16	64.2
Cayenne 4	12122018	21.542	18	70.0
Cayenne 5	21122018	80.248	17	80.0

**TABLE A3:** Microbiological evaluation.

Hazard	Results	Maximum permissible
Aerobic colony counts or *aerobic mesophylls*		500 cfu/g
Miel	200	
Esmeralda	230	
Cayenne 01	80	
Cayenne 02	80	
Cayenne 03	90	
Cayenne 04	75	
Cayenne 05	60	
*Coliforms* organisms		25 cfu/g
Miel	<0.3 m.s.	
Esmeralda	<0.3 m.s.	
Cayenne 01	<0.3 m.s.	
Cayenne 02	<0.3 m.s.	
Cayenne 03	<0.3 m.s.	
Cayenne 04	<0.3 m.s.	
Cayenne 05	<0.3 m.s.	
Yeasts and moulds		300 cfu/g
Miel	40 e.v.	
Esmeralda	100	
Cayenne 01	50 e.v.	
Cayenne 02	50 e.v.	
Cayenne 03	120 e.v.	
Cayenne 04	50 e.v.	
Cayenne 05	30 e.v.	
*Salmonella* spp		Absent in 25 g
Miel	Absence	
Esmeralda	Absence	
Cayenne 01	Absence	
Cayenne 02	Absence	
Cayenne 03	Absence	
Cayenne 04	Absence	
Cayenne 05	Absence	

Abbreviations: e.v., estimated value; m.s., method sensitivity.

**TABLE A4:** Determination of moisture (%) in the batches of dehydrated pineapple.

Sample	°Brix	*a*_w_
Miel	11.100^ab^	0.494^a^
Esmeralda	12.650^a^	0.546^a^
Cayenne 1	12.367^ab^	0.454^b^
Cayenne 2	11.033^ab^	0.501^a^
Cayenne 3	12.833^a^	0.531^a^
Cayenne 4	11.667^ab^	0.418^a^
Cayenne 5	10.700^b^	0.482^a^

**TABLE A5:** Physicochemical results of the three selected pineapple samples, fresh, and dry product.

Samples	Vitamin C	Carbohydrates	Dietary	Nutritional value
	(mg/100 g)	(g sugar/100 g)	Fiber (%)	(kJ/100 g)
Fresh	19	11.6	0.96	232
Miel	199.4 c	70.79 b	7.18 c	–
Esmeralda	52.47 b	76.97 a	7.61 b	–
Cayenne	32.2 a	69.94 b	10.98 a	–
Average dried pineapple mix				1240

*Note*: The different letter (a,b,c) indicates that there is a statically significant difference between the samples in a column (*p* < .05)

**TABLE A6:** Attributes defined for flash profile evaluation.

Appearance	Texture	Odor	Flavor	Aftertaste
White cape	Adhesiveness	Caramelized	Acid	Bitter
Colour	Crunchy	Citric	Astringent	
Fibrous	Hardness	Sweet	Caramelized	
Homogeneity	Fracturability	Intensity	Sweet	
Brown tips	Rough	Pineapple	Intensity	
Toasted		Fermented	Pineapple	
		Toasted		

**TABLE A7:** Thermodynamic states (Figure A6) of the DGA 200 System.

States	Phase	kg/s	°C	Bara	Enthalpy (kJ/kg)	Entropy (kJ/kgK)
01	Liquid	1.70	142.00	3.87	597.75	1.760
02	Liquid	1.70	72.93	3.87	305.63	0.991
03	Liquid	1.70	72.93	4.00	305.64	0.991
04	Liquid	1.70	95.00	3.96	398.33	1.250
05	Liquid	1.70	115.00	3.91	482.75	1.473
06	Gas	1.67	180.00	9.91	2778.15	6.591
07	Liquid	1.67	153.50	9.81	647.58	1.877
08	Liquid	1.67	133.69	9.77	562.59	1.673
09	Liquid	1.67	105.70	9.67	443.85	1.370
10	Gas	12.30	23.00	1.10	96.56	0.339
11	Gas	12.30	67.00	1.08	280.56	0.918

**TABLE A8:** Subsystems that make up the DGA 200.

Subsystems	Description
Geothermal steam pipe	The one used by the DGA 200 is in the separation platform No. 2 of the Domo San Pedro geothermal field (DSP), in Nayarit, Mexico. It has a pressure of 9.9 (Barg) at 180 (°C) and 20.8 (kg/s), of which only 8% is used to feed the dehydrator
Heat exchange system	The piping system is represented in thermodynamic states 6, 7, 8, and 9, see Figure A7. The design criteria were based on Reference Standards (NRF) used by Petróleos Mexicanos (PEMEX) and ASTM and ASME standards
Geothermal fluid transport system	The DGA has two systems, one is water–water, which serves to transfer heat from geothermal water to common intake water, which is not corrosive or fouling, and is made up of three HPE (see Figure A7: EA‐01A, EA‐01B and EA‐01C). The second working fluid is common intake water, and with this the air that will come into contact with the food is heated. The objective of the HPE is to protect the second heat exchange system (EA‐02) from corrosion and incrustations since the geothermal fluid has high concentrations of silica (SiO_2_)
Dehydrator	It is a maritime container of standard measures (2.5 × 2.9 × 12.1 m^3^), with axial air flow. It takes 12.3 kg/s from the environment that heats up to 90°C and enters it at a speed of 1.3–5 m/s. It has a diagonal design in the distribution duct that guarantees homogeneous air distribution within the oven. Results are reserved for presentation in another publication

**TABLE A9:** Descriptive table of the external colour of the SAGARPA pineapple (2005).

Num.	Peel Color Indices (PCI)	Description
1	Green	Skin is dark, with green tones and it shown in the 100% percent of the surface
2	A quarter	Eyes are green but show widening and yellowing of grooves (11 a 25%)
3	A half	Around 26%–50% of the peel surface is yellow (half ripe)
4	Three‐quarters	Fit for immediate consumption, the peel surface is yellow (full ripe)
5	Overripe	Fit for immediate consumption. Full yellow (over mature)

**TABLE A10:** Stages of the dehydration process, from receipt to processing.

Item	Process	Description
1	Reception and selection of raw materials	An inspection was carried out to determine its quality and if it meets the specifications regarding health (absence of insect attacks, spoiled, or rotten fruit), variety and state of maturity (°Brix, texture, color, and pH)
2	Storage and weighing	After inspection, it is weighed and destined for storage or processing, as appropriate
3	Selection and classification	Bruised or fungal fruit is removed. The classification is made by size and state of maturity. The fruit should have a firm texture. The ripe fruit (whose pulp is very soft, that is, when you squeeze it with your fingers, they sink) is removed
4	Weighing	The exact amount that will enter the process is weighed to determine the yield of the fruit. The residues (peel and crown) were weighed
5	Washing and disinfection	The fruit is immersed in a tub of water for washing. This removes dust, dirt, and other foreign particles. The cleaned fruit is disinfected, soaked in a disinfectant solution for 5 min. After washing with chlorinated water, we proceed to wash with drinking water, to remove any residual chlorine that may have remained
6	Peel	Manual peeling is performed on tables and with 300 series stainless steel knives
7	Chop	The fruit was cut into pieces of equal thickness (10 mm). This helps control dehydration levels and contributes to the uniformity of the final product
8	On tray	Place the fruits on the trays these must not be superimposed, but well distributed
9	Dehydration	Control the temperature and drying time variables. The dehydration process ranges from 70 to 75°C
10	Cooling	Let it cool down to room temperature. Then, it is collected and stored in cellophane bags with lateral gussets and 25 μm (standard)
11	Inspection	The inspection is visual to be able to observe that there are no foreign materials in the product, such as hairs, fruit peels, and metals
12	Packing	They are packaged in presentations of 30–60
