# Supplementary material for: Geothermal food dehydrator system, operation and sensory analysis, and dehydrated pineapple quality
Source: Food Sci Nutr. 2023 Feb 8;11(11):6711–27. doi: 10.1002/fsn3.3249 (PMC10630830; doi:10.1002/fsn3.3249)
Supplement: Supplementary file 1 — Data S1 [file FSN3-11-6711-s005.docx]

# Supplementary material

## DGA 200 System

Moisture removal in food can be done by different methods, however 85% of dehydrators in the industry are hot air and direct heating with temperature ranges between 50 and 400 °C (of course this is general information, and the temperature does not go so high for food products). (Mujumdar et al., 2010) The DGA 200 is a technological development conceived at the National Autonomous University of Mexico (UNAM), it was installed and tested in the first private geothermal field in Mexico and its production capacity ranges from 150 to 200 dry kg/day, and for this reason it is considered the first technological development of this nature; Although there are already geothermal dehydrators in several countries that have this energy resource, most are developments at the prototype level and the quality of dehydrated foods has not been documented. Therefore, it was sought that the DGA 200 be at a level of commercial production under the quality standards demanded by the industry and at the same time meeting consumer expectations through a sensory analysis.

It could be thought that a food with a higher moisture content will represent a higher energy consumption for the process, however, there are studies that show that the content of dissolved solids influences the energy consumption required in the process, and pineapple is precisely one of the food that needs more energy to dehydrate it (Hossain et al., 2001; Simal et al., 2006); and for this reason it was used as a reference feed in the validation of the DGA 200. Table S7 and Fig. S6 show the thermodynamic states of the process and the Process Flow Diagram, respectively, of the dehydrator system. Dehydration system is characterized by four subsystems 1) Geothermal steam pipe, 2) Heat exchange system, 3) Geothermal fluid transport system and 4) Dehydration chamber, in Table S8 each one is defined.

# Tables and Figures

Table S1 Guidance of bacterial pathogens (the Hazard) specifications for dehydrated pineapple.

| **Hazard** | **Maximum permissible** |
| --- | --- |
| Aerobic Colony Counts or *aerobic mesophylls* | 500 cfu/g |
|  |  |
| *Coliforms* organisms | 25 cfu/g |
|  |  |
| Yeasts and moulds | 300 cfu/g |
|  |  |
| *Staphylococcus* *aureus* (coagulase-positive) | < 100 cfu/g |
|  |  |
| *Salmonella* *spp* | Absent in 25 g |
| *Escherichia* *coli* | Negative |
|  |  |
| † cfu: colony forming unit | |
|  |  |
|  |  |

Table S2 Information on the seven batches processed in the DGA 200.

| **Pineapple** | **Production**  **lot** | **kg dry matter** | **Drying time**  **hours.** | **Temperature °C** |  |
| --- | --- | --- | --- | --- | --- |
| Miel | 08122018 | 14.300 | 15 | 78.4 | |
| Esmeralda | 11122018 | 20.078 | 20 | 67.1 | |
| Cayenne 1 | 15122018 | 31.528 | 18 | 71.4 | |
| Cayenne 2 | 19122018 | 36.078 | 18 | 72.0 | |
| Cayenne 3 | 09122018 | 10.482 | 16 | 64.2 | |
| Cayenne 4 | 12122018 | 21.542 | 18 | 70.0 | |
| Cayenne 5 | 21122018 | 80.248 | 17 | 80.0 | |
|  |  |  |  |  | |


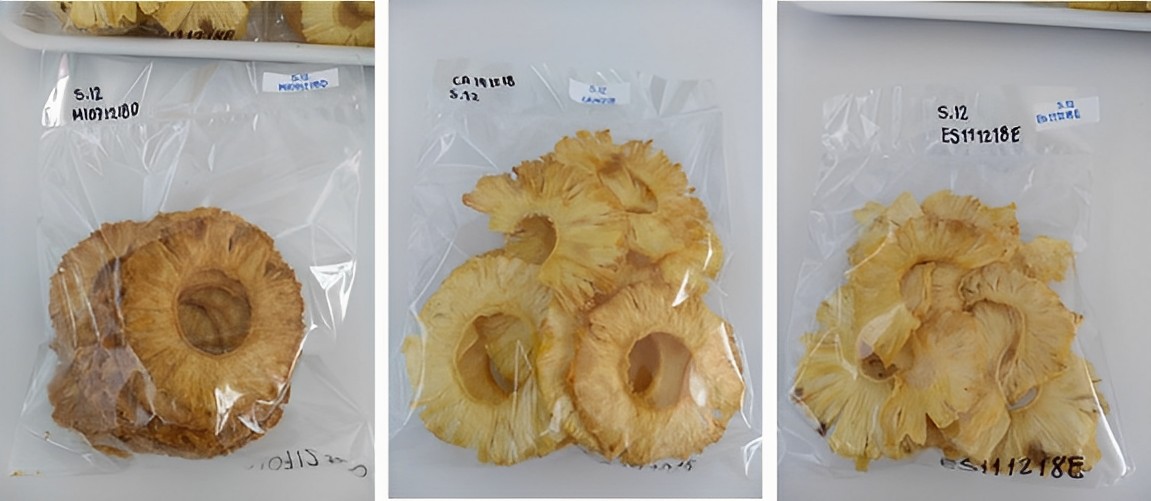


Fig. S1 Dried pineapple, from left to right Miel, Cayenne 2, and Esmeralda.

Table S3 Microbiological evaluation.

| **Hazard** | **Results** | **Maximum permissible** |
| --- | --- | --- |
| Aerobic Colony Counts or *aerobic mesophylls* |  | 500 cfu/g |
| Miel | 200 |  |
| Esmeralda | 230 |  |
| Cayenne 01 | 80 |  |
| Cayenne 02 | 80 |  |
| Cayenne 03 | 90 |  |
| Cayenne 04 | 75 |  |
| Cayenne 05 | 60 |  |
| *Coliforms* organisms |  | 25 cfu/g |
| Miel | <0.3 m.s. |  |
| Esmeralda | <0.3 m.s. |  |
| Cayenne 01 | <0.3 m.s. |  |
| Cayenne 02 | <0.3 m.s. |  |
| Cayenne 03 | <0.3 m.s. |  |
| Cayenne 04 | <0.3 m.s. |  |
| Cayenne 05 | <0.3 m.s. |  |
| Yeasts and moulds |  | 300 cfu/g |
| Miel | 40 e.v. |  |
| Esmeralda | 100 |  |
| Cayenne 01 | 50 e.v. |  |
| Cayenne 02 | 50 e.v. |  |
| Cayenne 03 | 120 e.v. |  |
| Cayenne 04 | 50 e.v. |  |
| Cayenne 05 | 30 e.v. |  |
| *Salmonella spp* |  | Absent in 25 g |
| Miel | Absence |  |
| Esmeralda | Absence |  |
| Cayenne 01 | Absence |  |
| Cayenne 02 | Absence |  |
| Cayenne 03 | Absence |  |
| Cayenne 04 | Absence |  |
| Cayenne 05 | Absence |  |
| † e.v.: estimated value  ‡ m.s.: method sensitivity |  |  |

Table S4 Determination of moisture (%) in the batches of dehydrated pineapple.

| **Sample** | **°Brix** | **aw** |
| --- | --- | --- |
| Miel | 11.100^ab^ | 0.494^a^ |
| Esmeralda | 12.650^a^ | 0.546^a^ |
| Cayenne 1 | 12.367^ab^ | 0.454^b^ |
| Cayenne 2 | 11.033^ab^ | 0.501^a^ |
| Cayenne 3 | 12.833^a^ | 0.531^a^ |
| Cayenne 4 | 11.667^ab^ | 0.418^a^ |
| Cayenne 5 | 10.700^b^ | 0.482^a^ |

Table S5 Physicochemical results of the three selected pineapple samples, fresh and dry product.

| **Samples** | **Vitamin C** | **Carbohydrates** | **Dietary** | **Nutritional value** |  |
| --- | --- | --- | --- | --- | --- |
|  | **[mg/100 g]** | **[g sugar/100 g]** | **Fiber (%)** | **[kJ/100g]** |  |
| Fresh | 19 | 11.6 | 0.96 | 232 |  |
| Miel | 199.4c | 70.79b | 7.18c | --- |  |
| Esmeralda | 52.47b | 76.97a | 7.61b | --- |  |
| Cayenne | 32.2a | 69.94b | 10.98a | --- |  |
| **Average dried pineapple mix** | | | | 1,240 |  |
| † a,b,c. The different letter indicates that there is a statically significant difference between the samples in a column (p<0.05) | | | | |  |
|  |  |  |  |  |  |

Table S6 Attributes defined for Flash profile evaluation.

| **Appearance** | **Texture** | **Odour** | **Flavour** | **Aftertaste** |
| --- | --- | --- | --- | --- |
| white cape | adhesiveness | caramelized | acid | bitter |
| colour | crunchy | citric | astringent |  |
| fibrous | hardness | sweet | caramelized |  |
| homogeneity | fracturability | intensity | sweet |  |
| brown tips | rough | pineapple | intensity |  |
| toasted |  | fermented | pineapple |  |
|  |  | toasted |  |  |


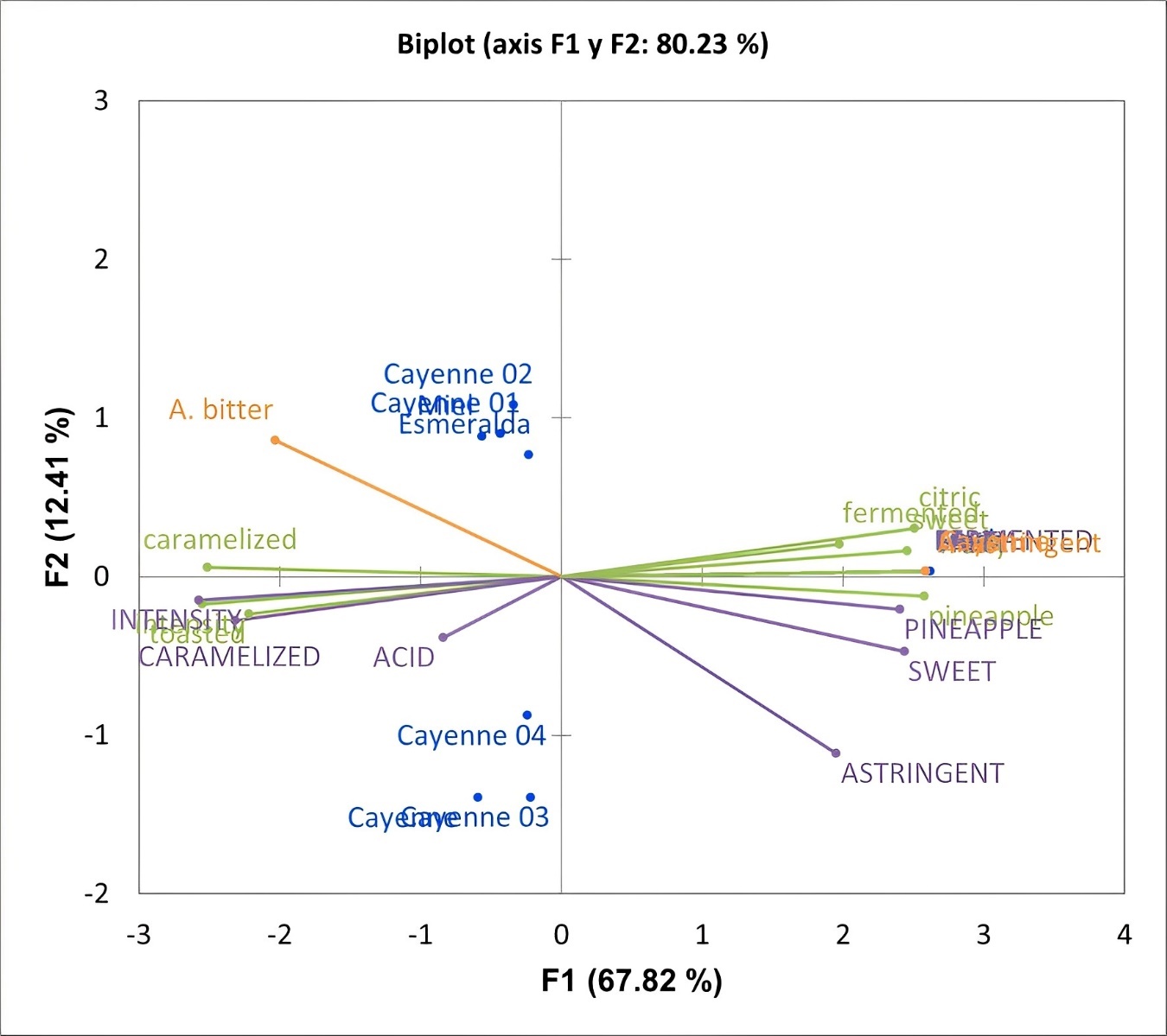


Fig. S2 Principal Component Analysis from the odour and taste comparison for fresh and dehydrated pineapple.


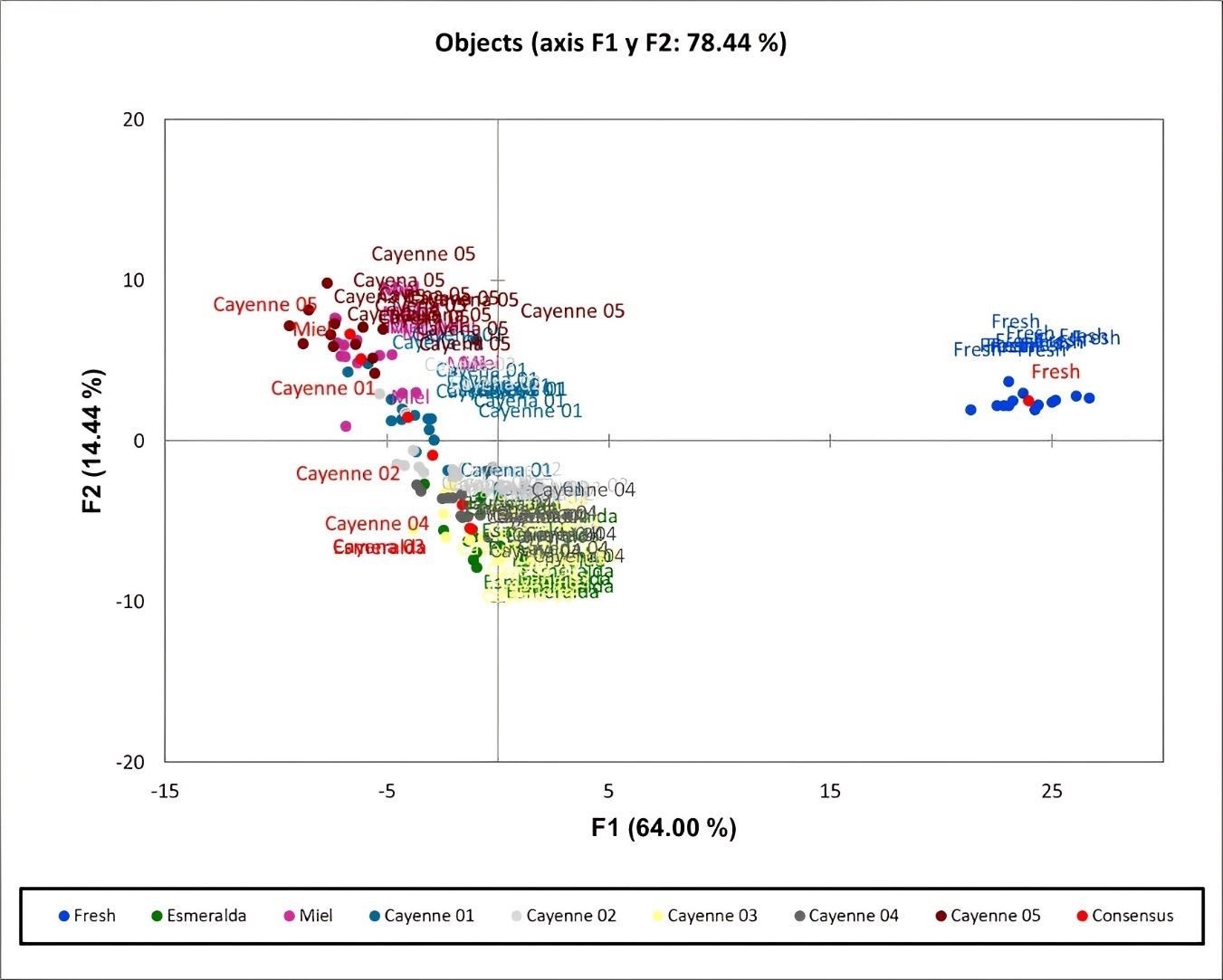


Fig. S3 Consensus result of the seven pineapple samples.


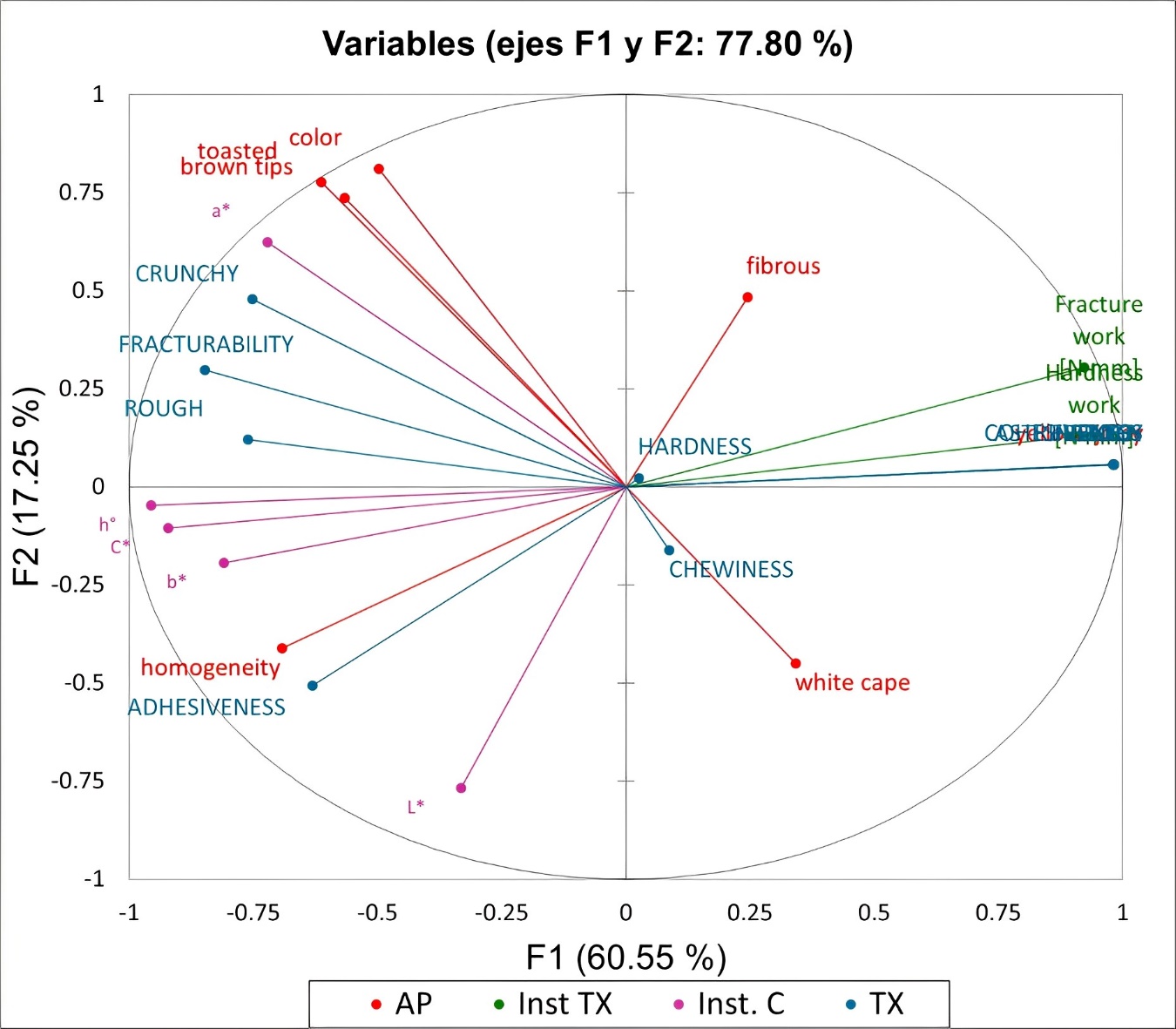


Fig. S4 PCA mapping from Multiple Factor Analysis of the instrumental and sensory (appearance and texture) in dehydrated pineapple.


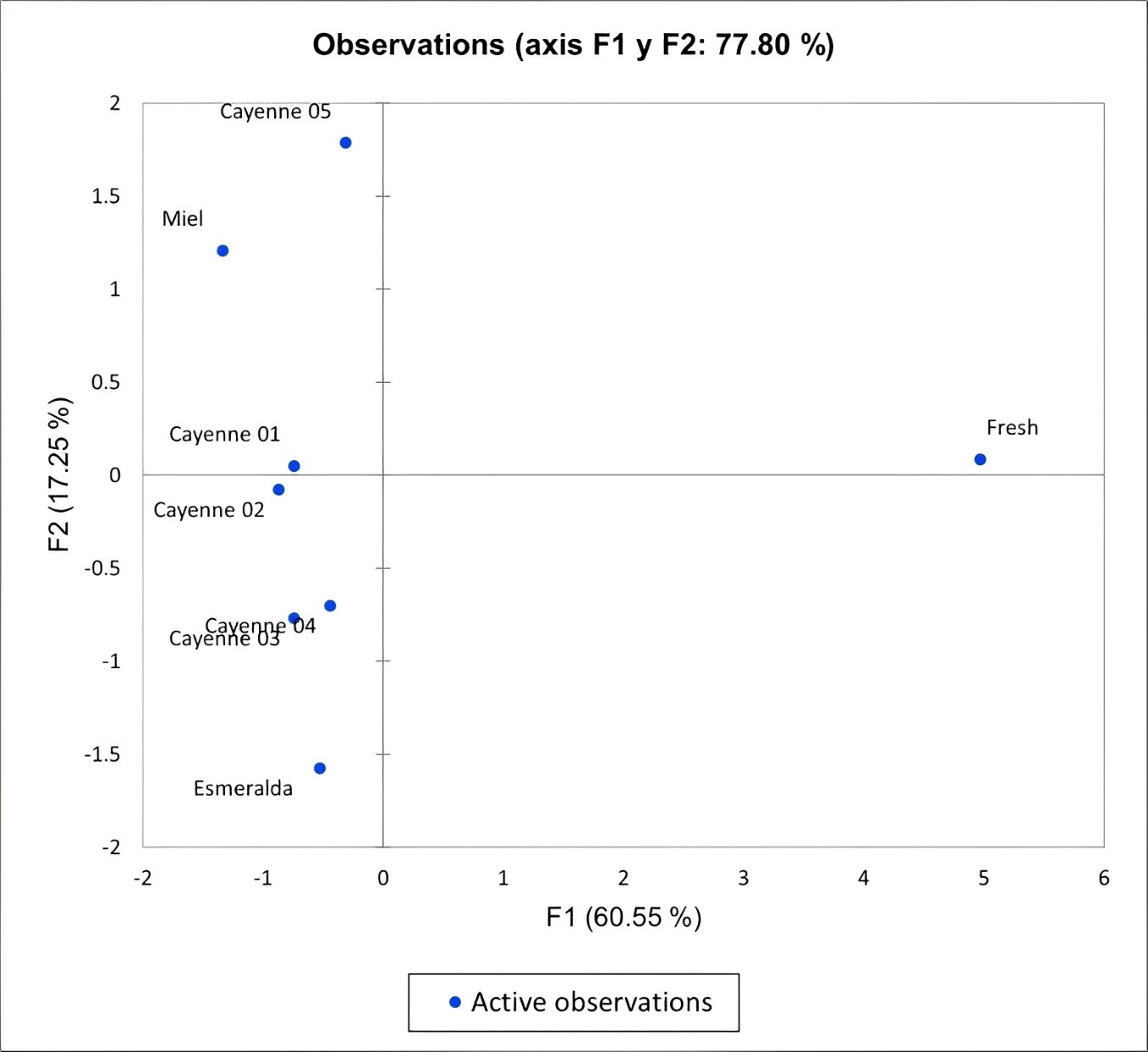


Fig. S5 Dehydrated pineapple sensory space, obtained from Multiple Factor Analysis.

Table S7 Thermodynamic states (Fig. 6) of the DGA 200 System.

| **States** | **Phase** | **kg/s** | **°C** | **Bara** | **Enthalpy**  **kJ/kg** | **Entropy**  **(kJ/kgK)** |
| --- | --- | --- | --- | --- | --- | --- |
| 01 | Liquid | 1.70 | 142.00 | 3.87 | 597.75 | 1.760 |
| 02 | Liquid | 1.70 | 72.93 | 3.87 | 305.63 | 0.991 |
| 03 | Liquid | 1.70 | 72.93 | 4.00 | 305.64 | 0.991 |
| 04 | Liquid | 1.70 | 95.00 | 3.96 | 398.33 | 1.250 |
| 05 | Liquid | 1.70 | 115.00 | 3.91 | 482.75 | 1.473 |
| 06 | Gas | 1.67 | 180.00 | 9.91 | 2778.15 | 6.591 |
| 07 | Liquid | 1.67 | 153.50 | 9.81 | 647.58 | 1.877 |
| 08 | Liquid | 1.67 | 133.69 | 9.77 | 562.59 | 1.673 |
| 09 | Liquid | 1.67 | 105.70 | 9.67 | 443.85 | 1.370 |
| 10 | Gas | 12.30 | 23.00 | 1.10 | 96.56 | 0.339 |
| 11 | Gas | 12.30 | 67.00 | 1.08 | 280.56 | 0.918 |


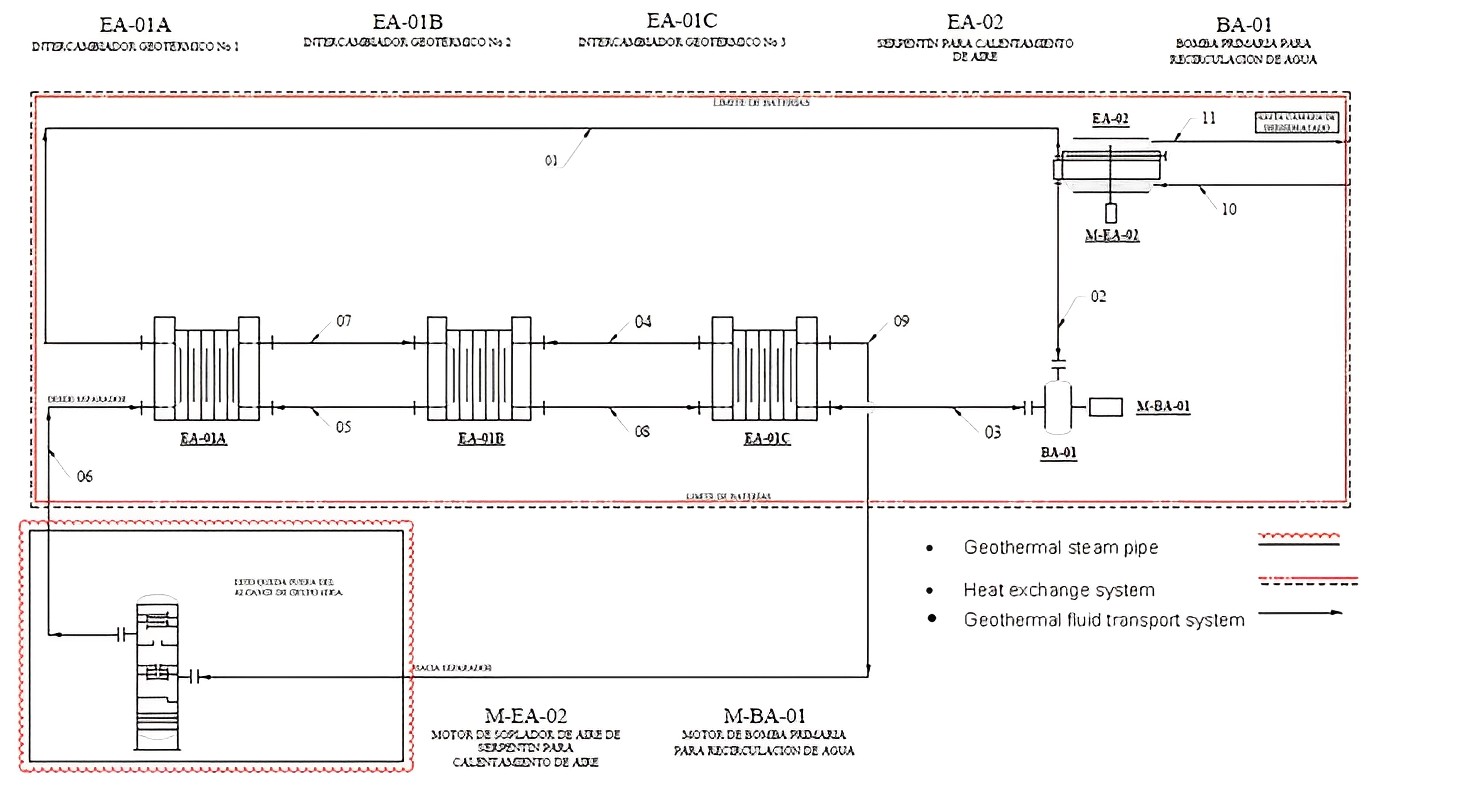


Fig. S6 DGA 200 System Process Flow Diagram.

Table S8 Subsystems that make up the DGA 200.

| **Subsystems** | **Description** |
| --- | --- |
| Geothermal steam pipe | The one used by the DGA 200 is in the separation platform No. 2 of the Domo San Pedro geothermal field (DSP), in Nayarit, Mexico. It has a pressure of 9.9 [Barg] at 180 [°C] and 20.8 [kg/s], of which only 8% is used to feed the dehydrator. |
|  |  |
| Heat Exchange system | The piping system is represented in thermodynamic states 6, 7, 8 and 9, see Fig. S7. The design criteria were based on Reference Standards (NRF) used by **Petróleos Mexicanos** (PEMEX) and ASTM and ASME standards. |
|  |  |
| Geothermal fluid transport system | The DGA has two systems, one is water-water, which serves to transfer heat from geothermal water to common intake water, which is not corrosive or fouling, and is made up of three HPE (see Fig. S7: EA-01A, EA-01B and EA-01C). The second working fluid is common intake water, and with this the air that will come into contact with the food is heated. The objective of the HPE is to protect the second heat exchange system (EA-02) from corrosion and incrustations since the geothermal fluid has high concentrations of silica (SiO_2_). |
|  |  |
| Dehydrator | It is a maritime container of standard measures (2.5 x 2.9 x 12.1 m^3^), with axial air flow. It takes 12.3 kg/s from the environment that heats up to 90 °C and enters it at a speed of 1.3 to 5 m/s. It has a diagonal design in the distribution duct that guarantees homogeneous air distribution within the oven. Results are reserved for presentation in another publication. |


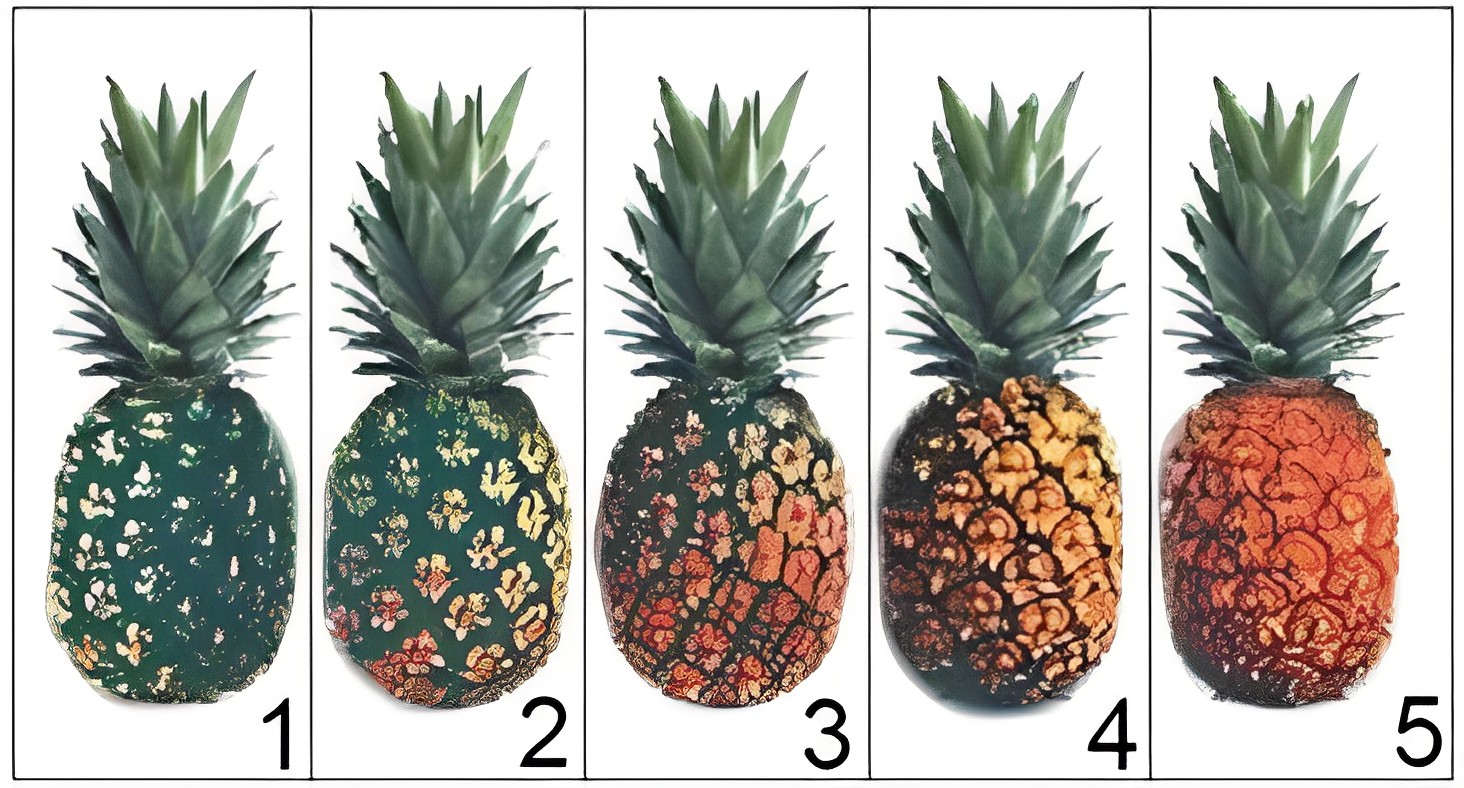


Fig. S7 Table for the classification of pineapple according to external colour, (SAGARPA, 2005).

Table S9 Descriptive table of the external colour of the SAGARPA pineapple (2005).

| **Num.** | **Peel Colour Indices**  **PCI** | **Description** |
| --- | --- | --- |
| 1 | green | Skin is dark, with green tones and it shown in the 100% percent of the surface. |
| 2 | a quarter | Eyes are green but show widening and yellowing of grooves (11 a 25%). |
| 3 | a half | About 26 to 50% of the peel surface is yellow (half ripe). |
| 4 | three-quarters | Fit for immediate consumption, the peel surface is yellow (full ripe). |
| 5 | overripe | Fit for immediate consumption. Full yellow (over mature). |

Table S10 *Stages of the dehydration process, from receipt to processing.*

| **ITEM** | **Process** | **Description** |
| --- | --- | --- |
| 1 | Reception and selection of raw materials. | An inspection was carried out to determine its quality and if it meets the specifications regarding health (absence of insect attacks, spoiled or rotten fruit), variety and state of maturity (°Brix, texture, colour, pH) |
| 2 | Storage and weighing | After inspection, it is weighed and destined for storage or processing, as appropriate. |
| 3 | Selection and classification | Bruised or fungal fruit is removed. The classification is made by size and state of maturity. The fruit should have a firm texture. The ripe fruit (whose pulp is very soft, that is, when you squeeze it with your fingers, they sink) is removed. |
| 4 | Weighing | The exact amount that will enter the process is weighed to determine the yield of the fruit. The residues (peel and crown) were weighed. |
| 5 | Washing and disinfection | The fruit is immersed in a tub of water for washing. This removes dust, dirt, and other foreign particles. The cleaned fruit is disinfected, soaked in a disinfectant solution for 5 minutes. After washing with chlorinated water, we proceed to wash with drinking water, to remove any residual chlorine that may have remained. |
| 6 | Peel | Manual peeling is done on tables and with 300 series stainless steel knives. |
| 7 | Chop | The fruit was cut into pieces of equal thickness (10 mm). This helps control dehydration levels and contributes to the uniformity of the final product. |
| 8 | On tray | Place the fruits on the trays these must not be superimposed, but well distributed. |
| 9 | Dehydration | Control the temperature and drying time variables. The dehydration process ranges from 70 to 75 °C. |
| 10 | Cooling | Let it cool down to room temperature. Then, it is collected and stored in cellophane bags with lateral gussets and 25 microns (standard). |
| 11 | Inspection | The inspection is visual to be able to observe that there are no foreign materials in the product, such as hairs, fruit peels, metals, etc. |
| 12 | Packing | They are packaged in presentations of 30 to 60. |


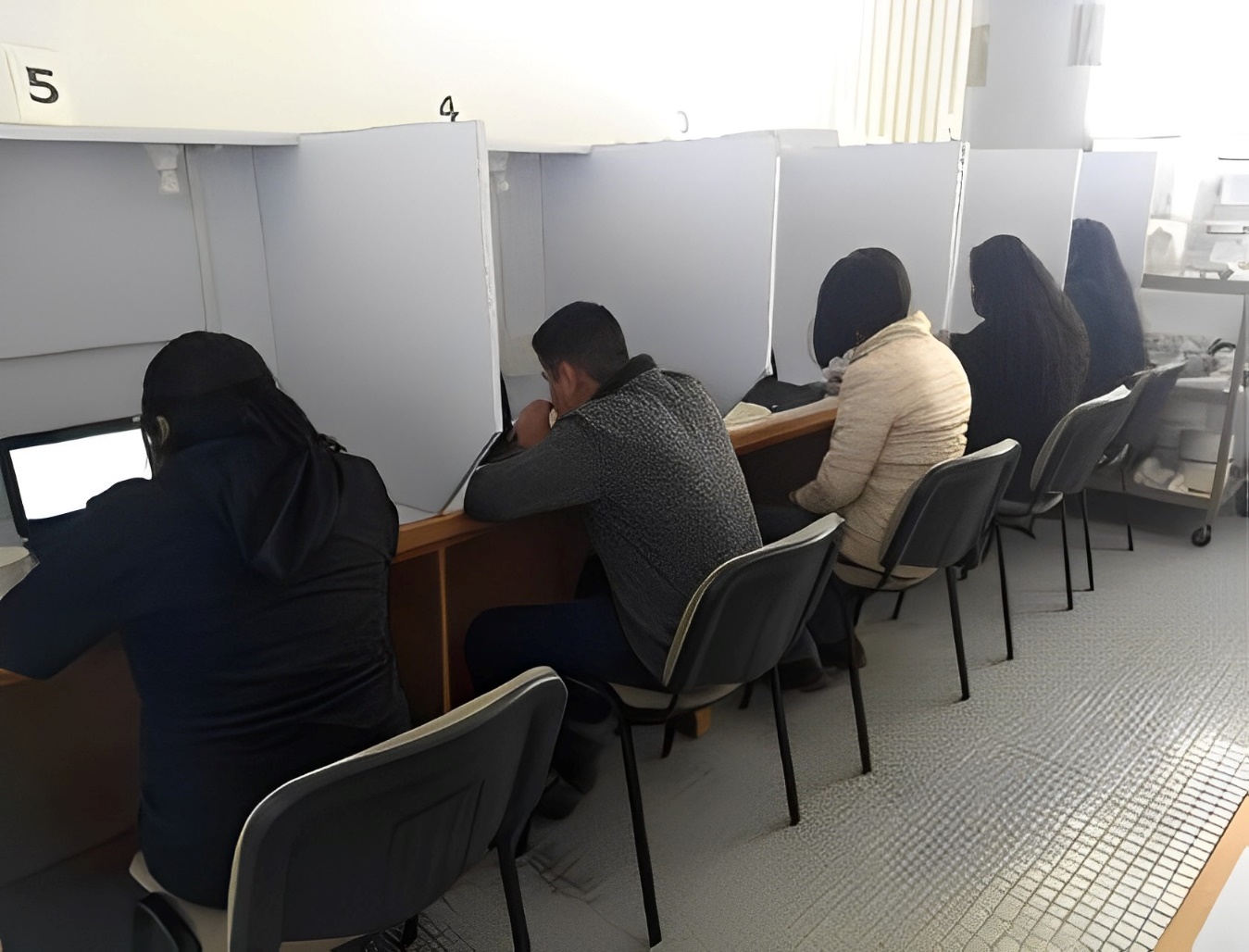


Fig. S8 Sensory evaluation of dehydrated pineapple.


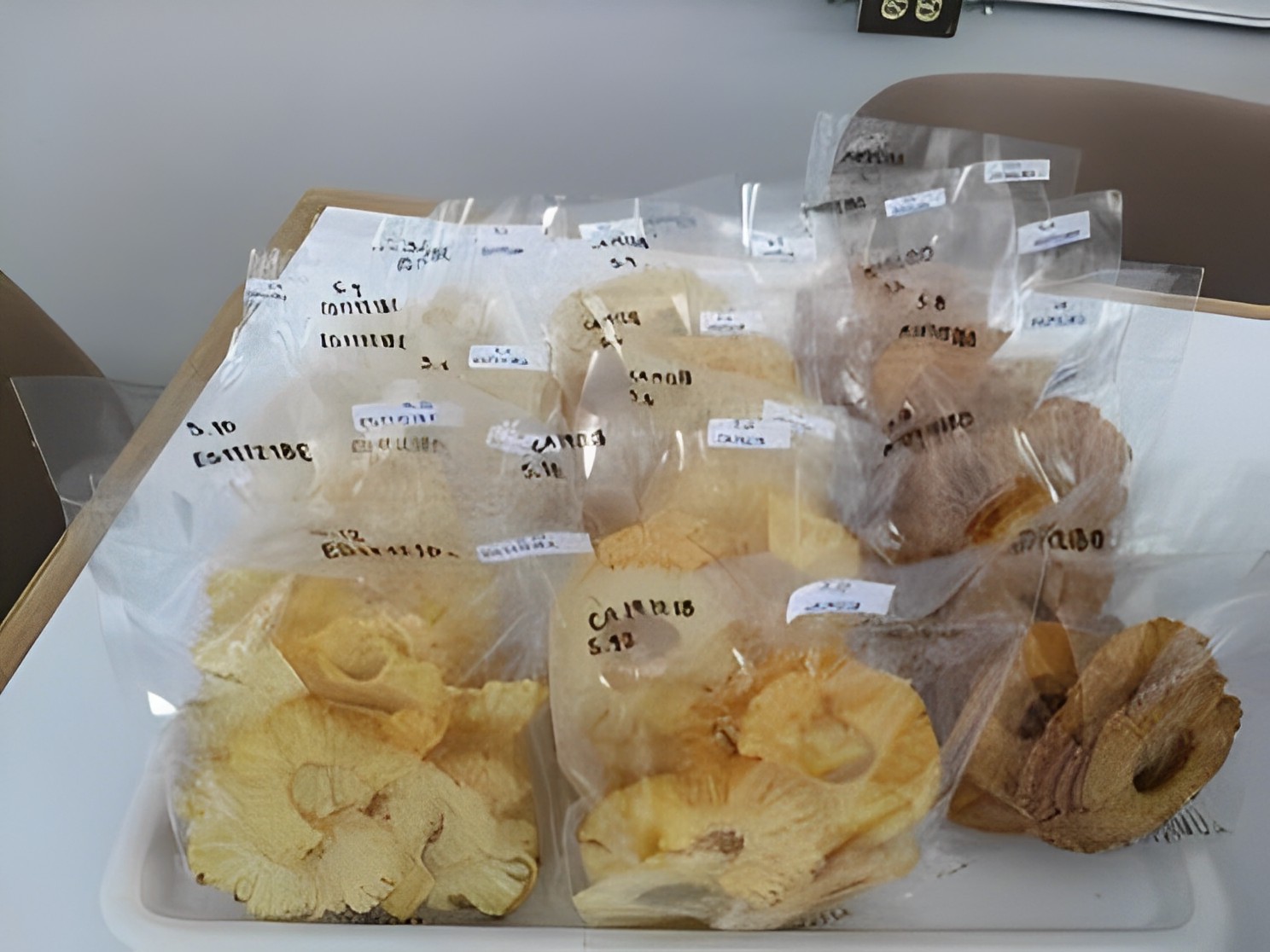


Fig. S9 Different batches of dehydrated pineapple.


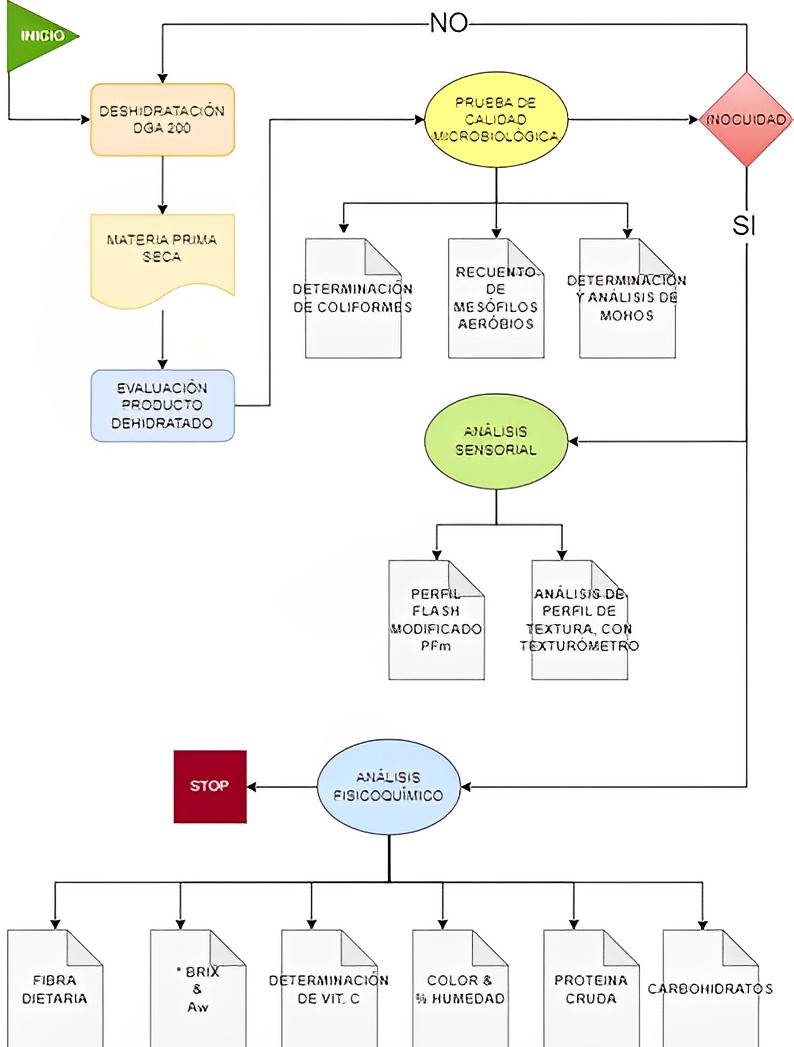


Fig. S10 Logic diagram of the research.
