# Supplementary figures and images for: Geothermal food dehydrator system, operation and sensory analysis, and dehydrated pineapple quality
Source: Food Sci Nutr. 2023 Feb 8;11(11):6711–27. doi: 10.1002/fsn3.3249 (PMC10630830; doi:10.1002/fsn3.3249)

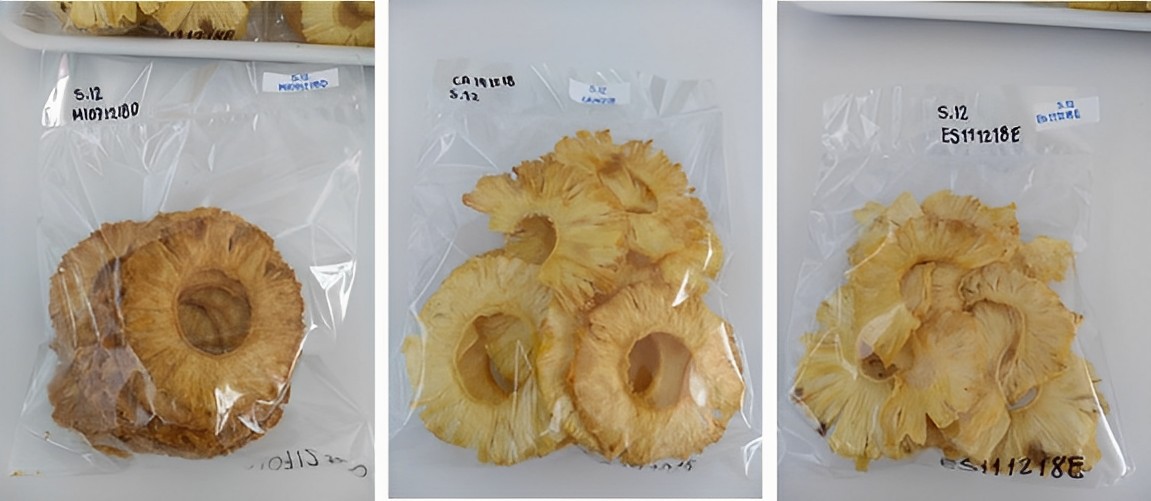

Supplement: Supplementary file 2 — Figure S1 [file FSN3-11-6711-s002.jpg]

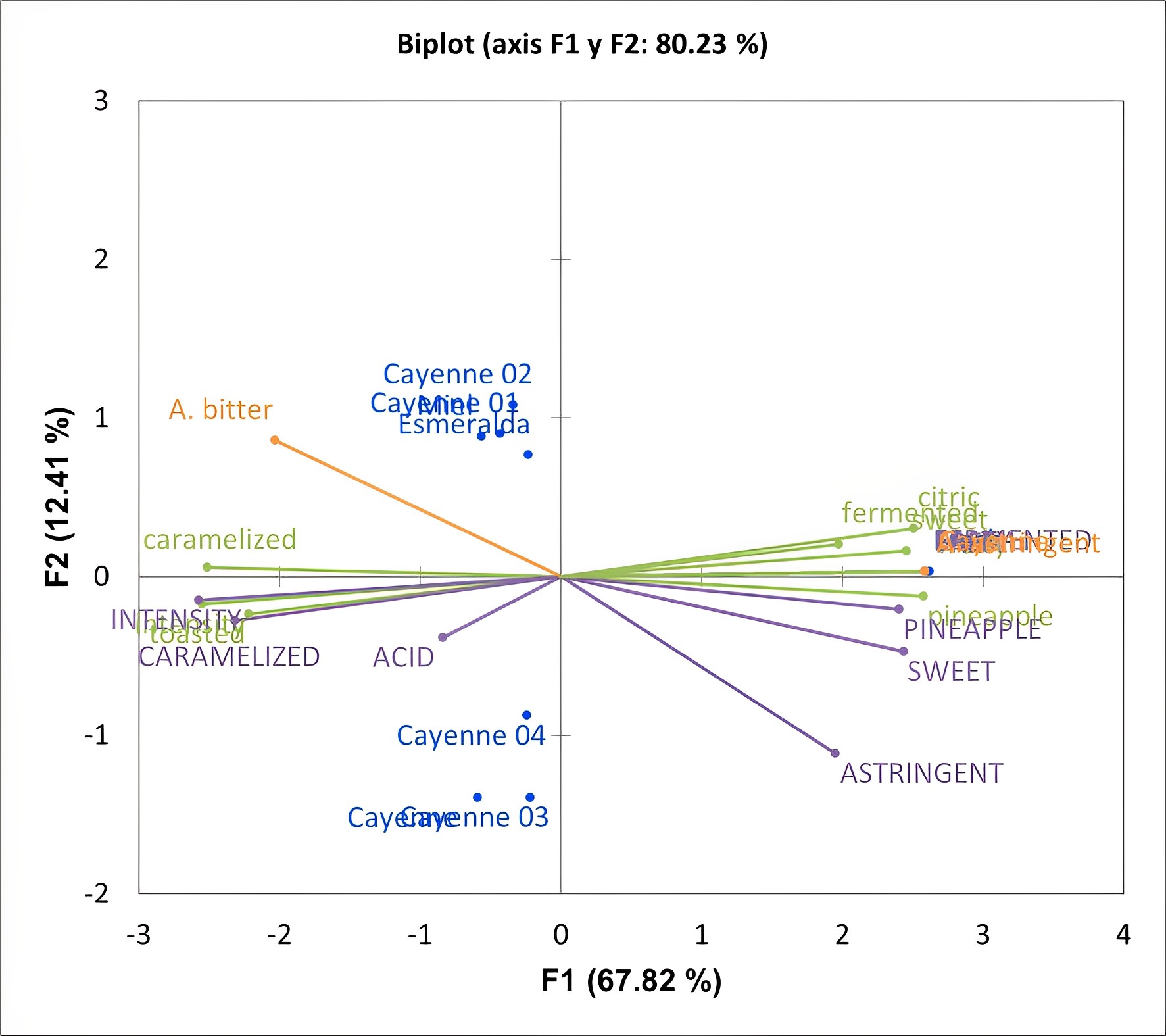

Supplement: Supplementary file 3 — Figure S2 [file FSN3-11-6711-s011.jpg]

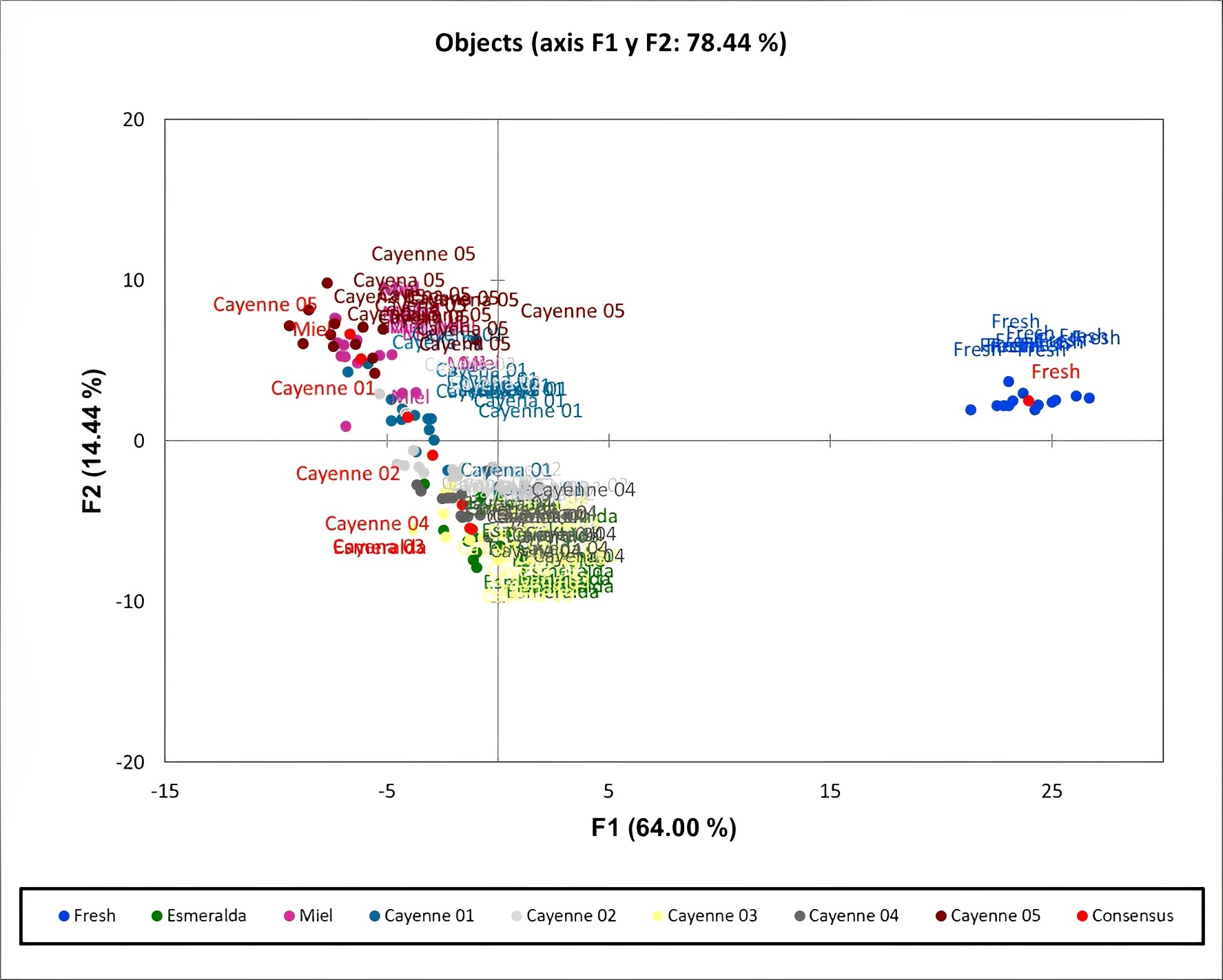

Supplement: Supplementary file 4 — Figure S3 [file FSN3-11-6711-s004.jpg]

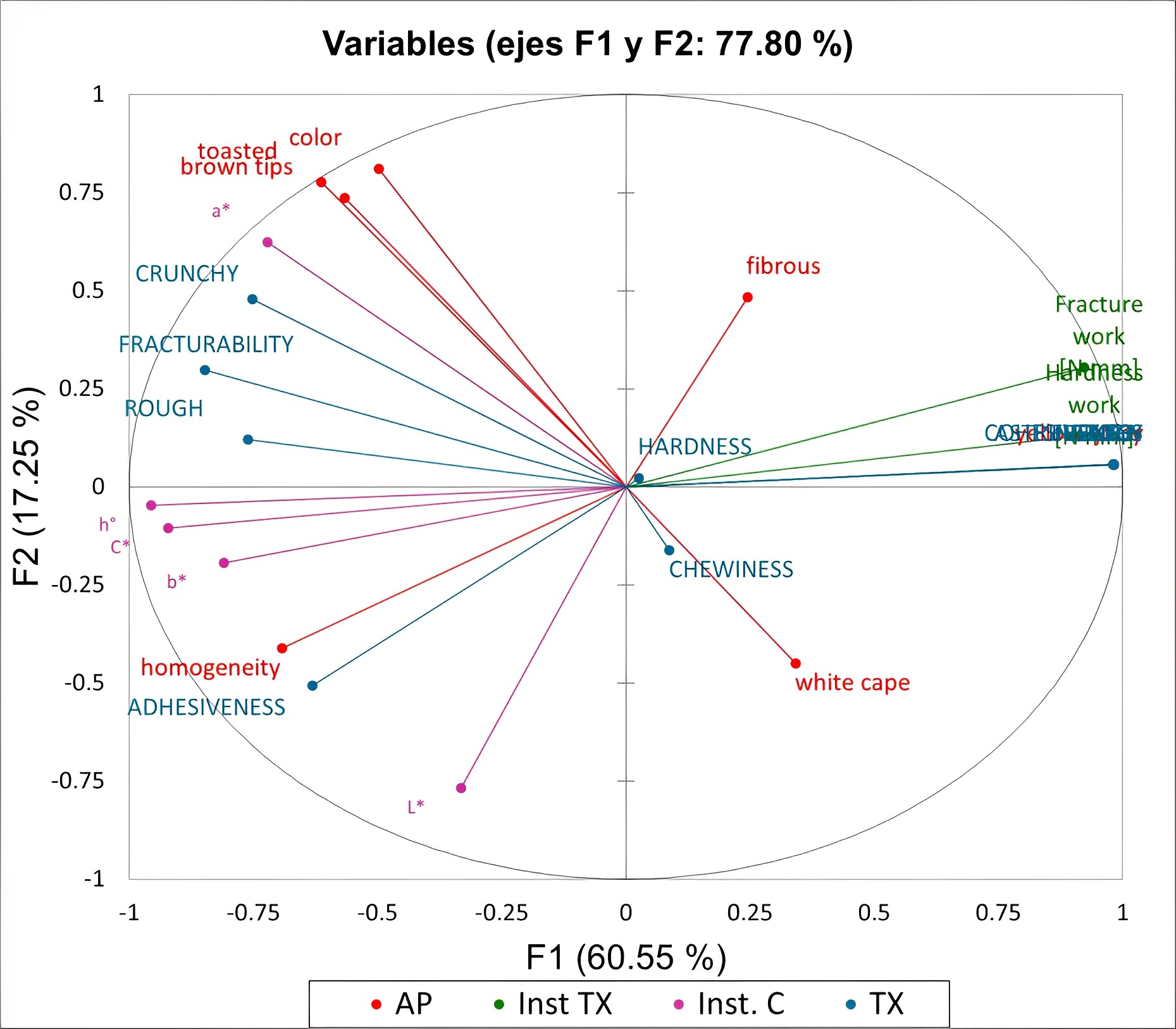

Supplement: Supplementary file 5 — Figure S4 [file FSN3-11-6711-s008.jpg]

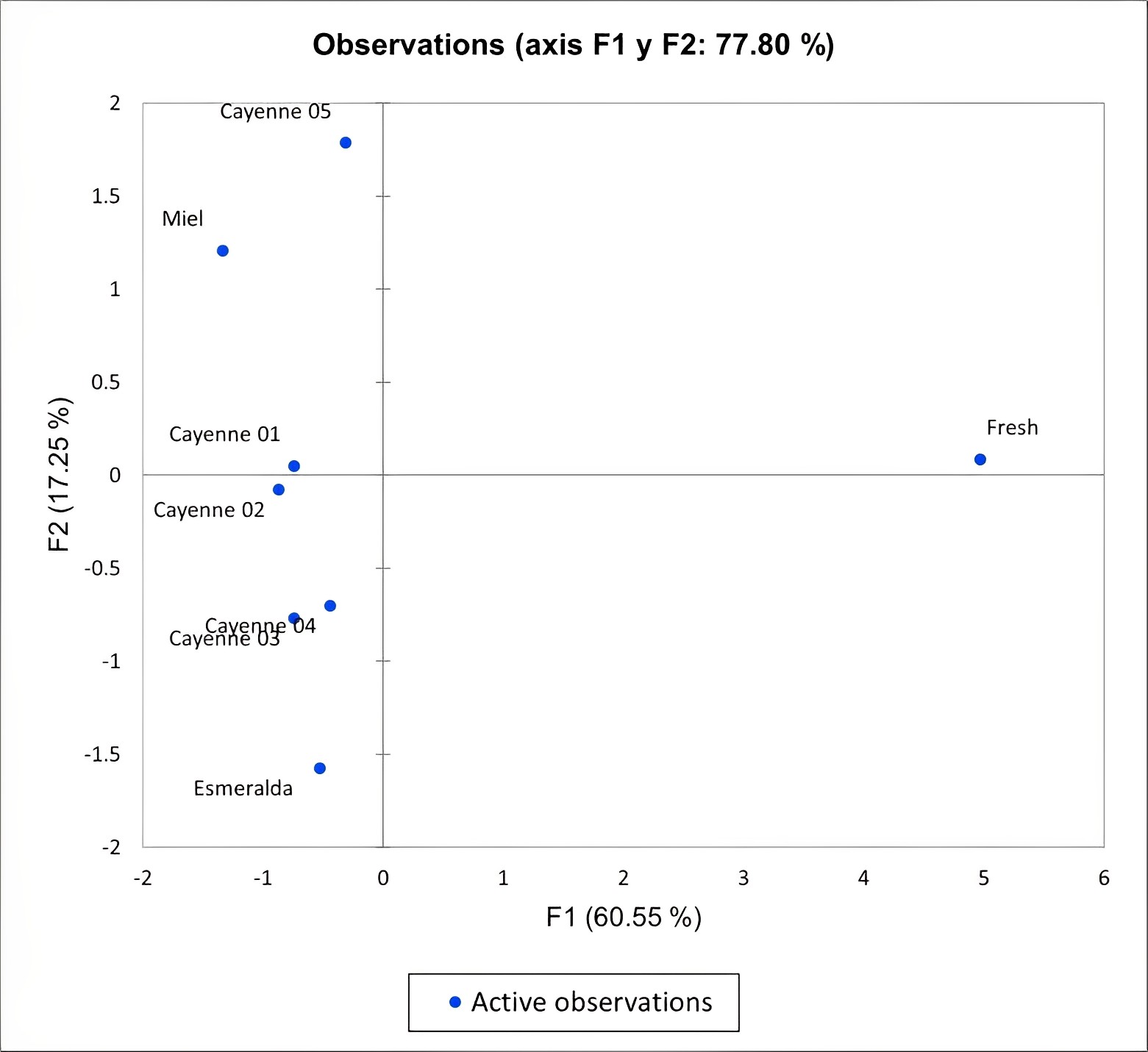

Supplement: Supplementary file 6 — Figure S5 [file FSN3-11-6711-s006.jpg]

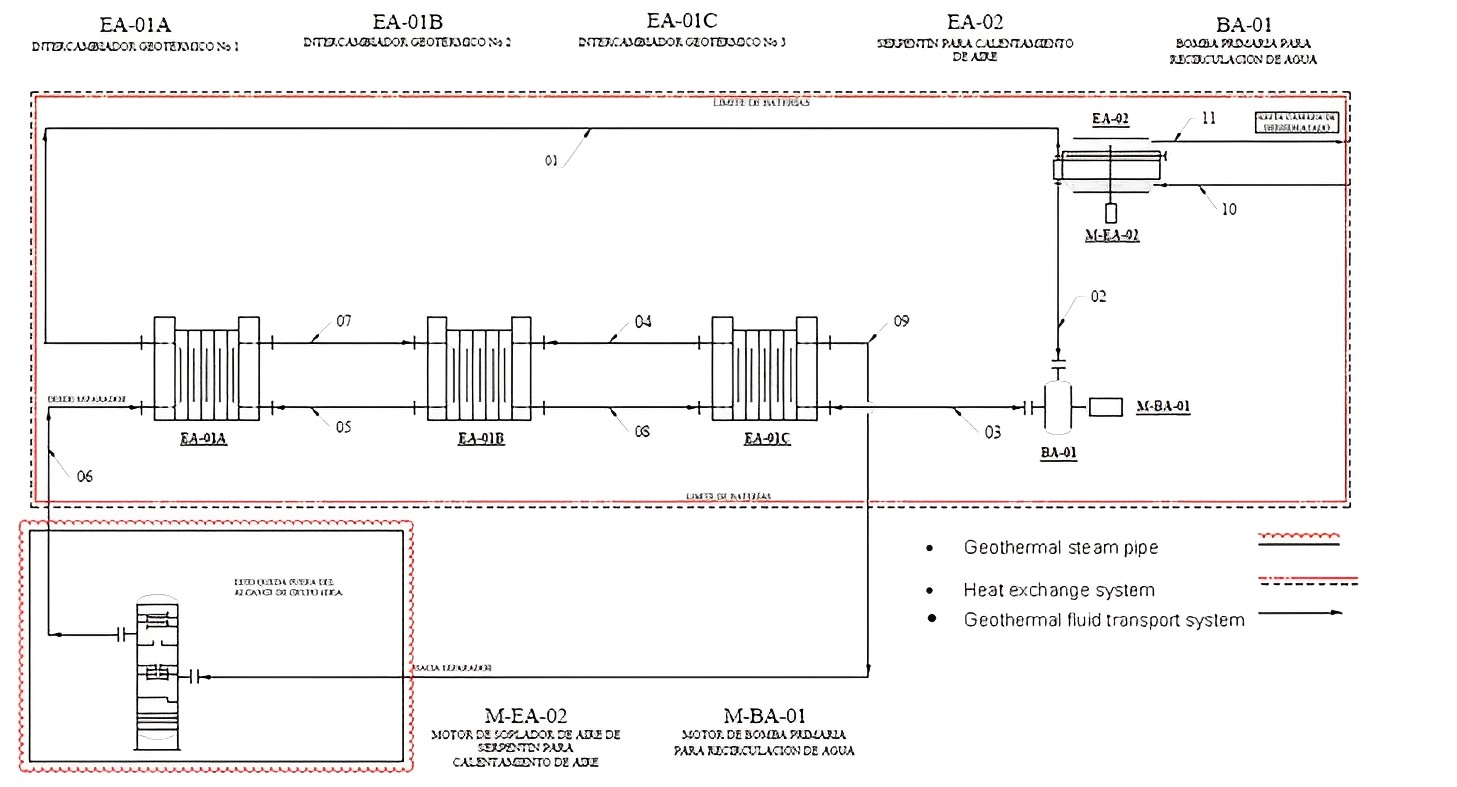

Supplement: Supplementary file 7 — Figure S6 [file FSN3-11-6711-s009.jpg]

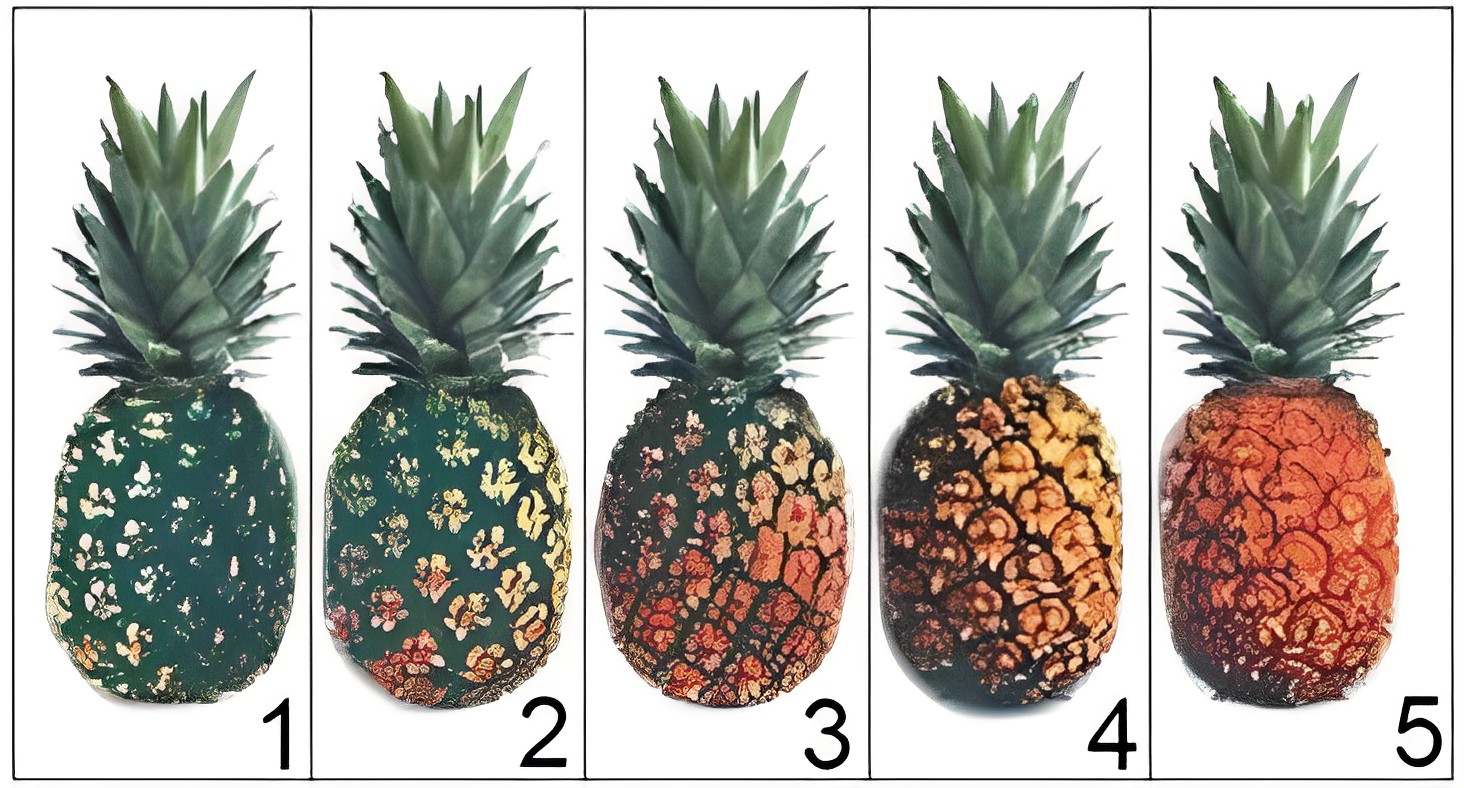

Supplement: Supplementary file 8 — Figure S7 [file FSN3-11-6711-s003.jpg]

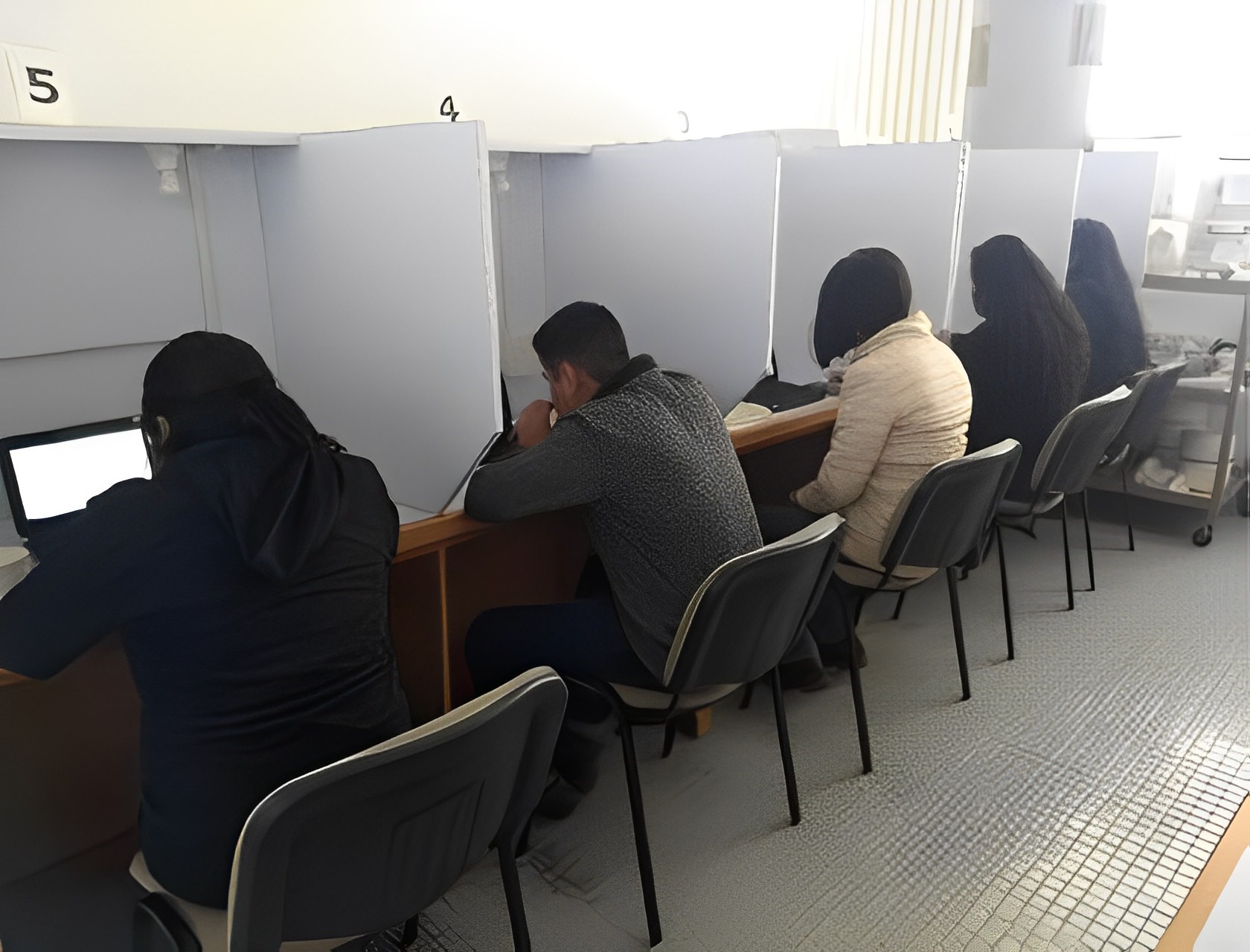

Supplement: Supplementary file 9 — Figure S8 [file FSN3-11-6711-s001.jpg]

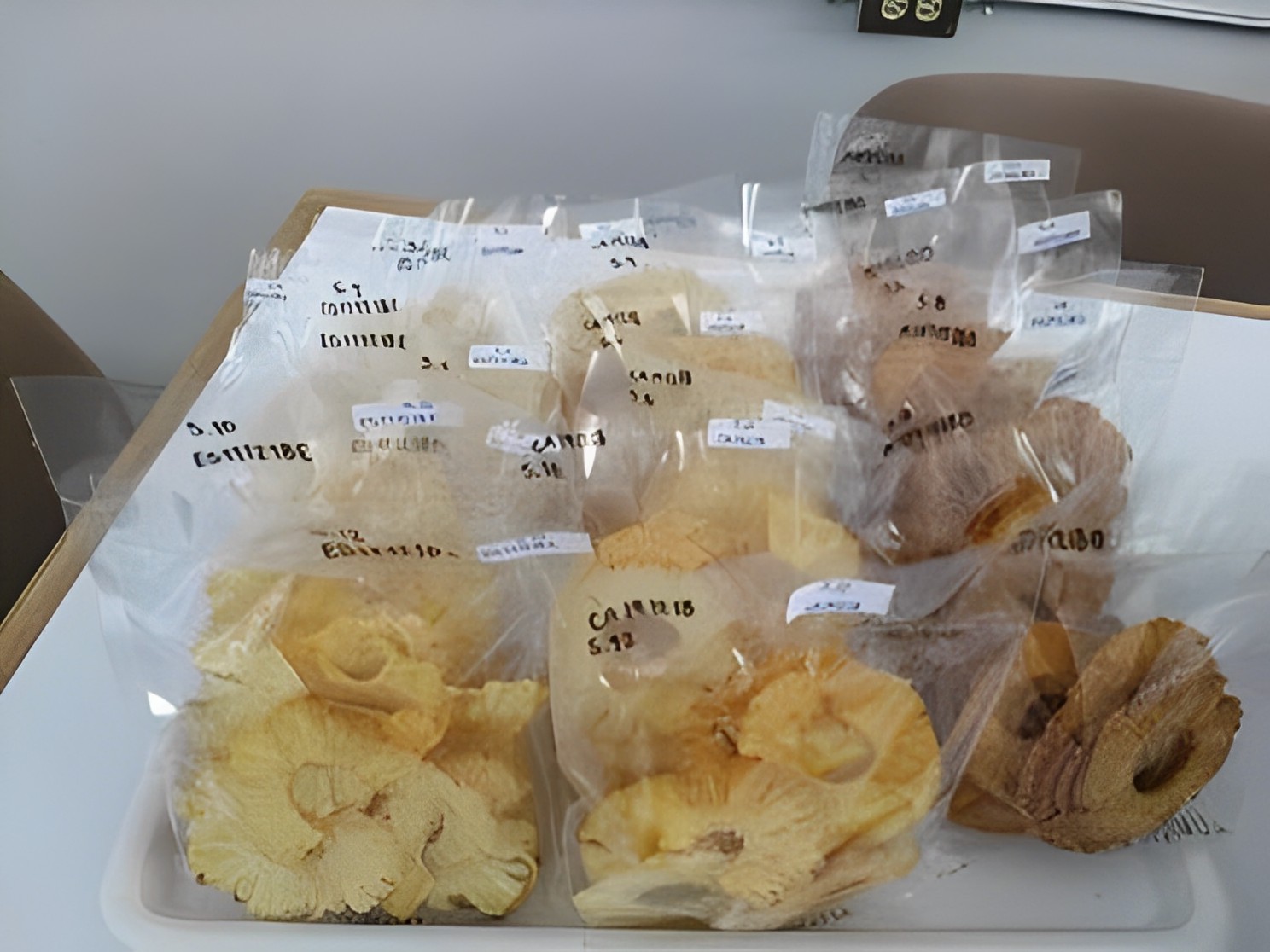

Supplement: Supplementary file 10 — Figure S9 [file FSN3-11-6711-s007.jpg]

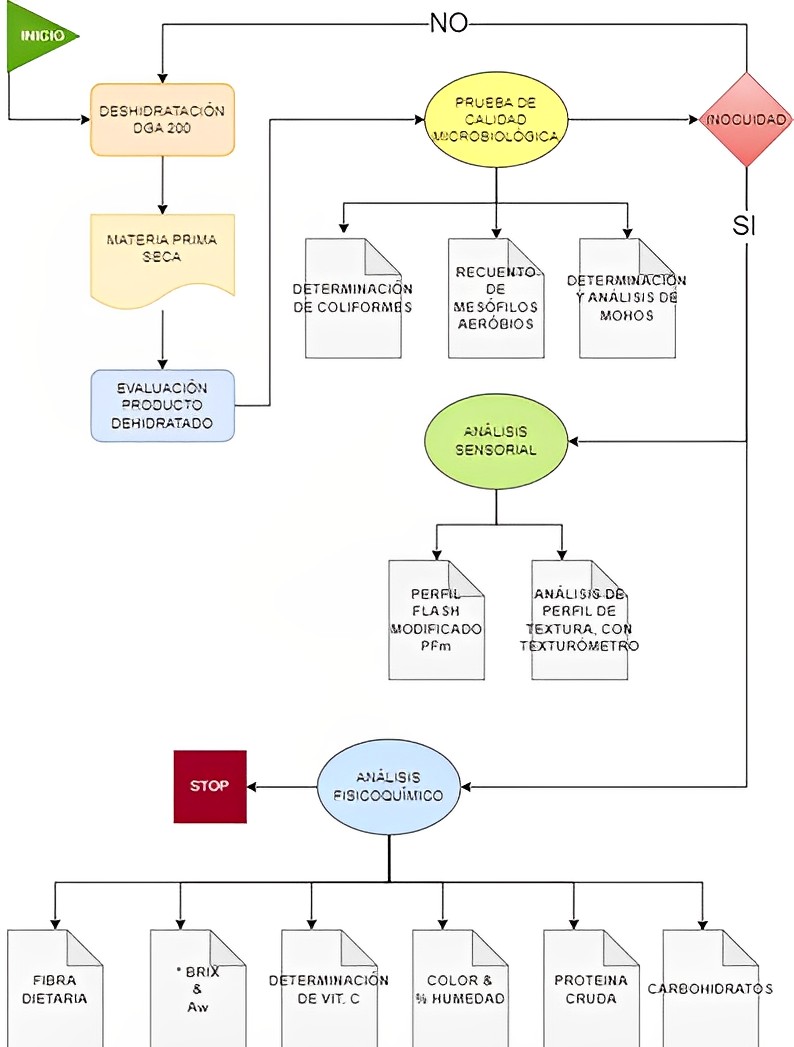

Supplement: Supplementary file 11 — Figure S10 [file FSN3-11-6711-s010.jpg]
